# Complementary immunoregulatory effects of *Bifidobacterium longum* 1714^TM^ associated exopolysaccharide and tryptophan metabolism

**DOI:** 10.1016/j.crmicr.2025.100481

**Published:** 2025-09-28

**Authors:** David Groeger, Lu Yao, Fergus Collins, Ida Søgaard Larsen, Hern-Tze Tina Tan, Selena Healy, Valentina Ambrogi, Karolina Tykwinska, Martin Schmidt, Patrick Golletz, Barry Kiely, Gerard Clarke, Timothy G. Dinan, Eileen F. Murphy, Liam O’Mahony

**Affiliations:** a Novonesis, Cork, Ireland; b Human Health Research, Novonesis, Hørsholm, Denmark; c APC Microbiome Ireland, UCC, Cork, Ireland; d School of Microbiology, UCC, Cork, Ireland; e Novozymes Berlin GmbH, Berlin, Germany; f Department of Psychiatry and Neurobehavioural Science, UCC, Cork, Ireland; g Department of Medicine, UCC, Cork, Ireland

**Keywords:** Bifidobacterium longum, Tryptophan, Immunoregulatory, Exopolysaccharides

## Abstract

Specific bifidobacterial strains can reduce aberrant inflammatory responses, but the strain-specific factors that promote regulatory immune responses are not well elucidated. The aims of this study were to assess the effects of *Bifidobacterium longum* 1714™ on innate inflammatory responses and to identify the strain-associated features that mediate its immunomodulatory effects. *B. longum* 1714 induced peripheral blood mononuclear cell (PBMC) and monocyte derived dendritic cell (MDDC) cytokine secretion and costimulatory molecule expression in a strain specific manner. *B. longum* 1714 consumption reduced excessive cytokine responses in LPS-induced, stress-induced and obesity-induced inflammation in murine models. *B. longum* 1714 produced a branched hexasaccharide repeating unit exopolysaccharide (EPS), which selectively induced PBMC secretion of IL-10. Furthermore, *B. longum* 1714 produced tryptophan and indole lactic acid (ILA) *in vitro*, and ILA directly reduced TLR-induced proinflammatory cytokine secretion and NF-κB activation. Finally, increased plasma levels of tryptophan and kynurenic acid were identified in humans following *B. longum* 1714 consumption.

*B. longum* 1714 influences the immune system through intrinsic features associated with strain specific surface EPS and via secreted metabolic products, which demonstrates the potential for multiple mechanisms through which a specific probiotic may modulate immune responses and potential neuroprotective pathways.

## Highlights

• *Bifidobacterium longum 1714 impacts the immune system via multiple mechanisms, reducing peripheral inflammation.*
• *B. longum 1714 reduced proinflammatory cytokine responses in stimulated PBMCS and in LPS-, stress-, and obesity-induced murine models.*
• *B. longum 1714 consumption in humans increased plasma tryptophan and kynurenic acid levels, supporting immunoregulatory effects.*
• *EPS and tryptophan metabolite ILA selectively promoted PBMC IL-10 secretion, while reducing TLR-induced cytokines and NF-κB activation.*
• *Intrinsic properties like its cell wall, EPS, and tryptophan metabolism contribute to immunomodulatory and anti-inflammatory effects.*

## Graphical abstract

**Figure:**
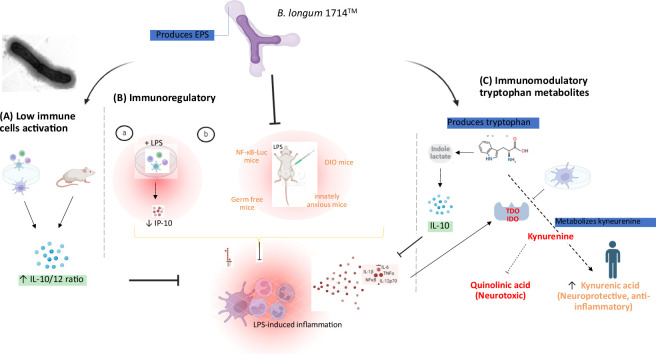
***Complementary immunoregulatory effects of Bifidobacterium longum 1714^TM^ associated exopolysaccharide and tryptophan metabolism***.

## Introduction

The gut microbiota contributes significantly to host health, and its composition and metabolic activity has profound effects on balancing pro-inflammatory immune responses and immune tolerance within mucosal tissue (Trompette et al., 2014; Frei et al., 2015).

Nearly 90 % of all immune cells in the body are associated with the gastrointestinal tract and these immune cells are continuously exposed to a wide range of microbes and microbial-derived compounds, with important systemic ramifications (Lee et al., 2011; Wu et al., 2010). Potent tolerance mechanisms should ensure that these immune cells do not over-react to non-pathogenic factors (*e.g.* commensal microbes), while maintaining the ability to respond to infectious challenges in a robust, effective and well controlled manner (Belkaid and Harrison, 2017; Littman and Rudensky, 2010; Zheng et al., 2020; Sivan et al., 2015). We and others have previously shown that specific microbes within the gut are required for efficient induction of immune tolerance networks. These immune effects are partially induced by activation of host pattern recognition receptors to microbial associated molecular patterns (MAMPs), but also immunoregulatory bacterial metabolites can trigger host G protein-coupled receptors (GPCRs), aryl hydrocarbon receptors (AhRs), nuclear hormone receptors such as the farnesoid X receptor, or can directly modulate gene expression through epigenetic mechanisms (Liwinski et al., 2020; Barcik et al., 2017; Forde et al., 2022; Hosseinkhani et al., 2021).

*Bifidobacterium* is a genus of particular interest as multiple studies have identified strains with immunomodulatory properties that impact gastrointestinal and systemic inflammation, while reduced levels of bifidobacteria in humans or experimental models were consistently associated with dysregulated immune responses (Gavzy et al., 2023; O'Mahony et al., 2008; O'Mahony et al., 2005; O'Mahony et al., 2006; Konieczna et al., 2012b; Groeger et al., 2013). One important pathway for bifidobacterial modulation of immune responses includes strain-specific cell surface structures such as exopolysaccharides (EPS) that can signal via TLR-2 (Altmann et al., 2016; Schiavi et al., 2016; Schiavi et al., 2018).

*Bifidobacterium longum* subsp. *longum 1714*™ has been evaluated in a number of preclinical animal models and human studies (Savignac et al., 2014; Savignac et al., 2015; Allen et al., 2016; Wang et al., 2019; Patterson et al., 2024). In a recent study in Irritable Bowel Syndrome (IBS) patients, *B. longum* 1714 in combination with *B. longum* 35624™ improved anxiety and depression which was associated with a decrease in the pro-inflammatory cytokine TNF-α (Groeger et al., 2023).While *B. longum* 1714 does not seem to significantly alter gut microbiota composition in these studies (Moloney et al., 2021), it’s not clear what direct impact (if any) *B. longum* 1714 has on the immune system, and if so, which mechanisms are employed by the strain for this interaction.

To address this, we assessed if the *in vitro* human immune response to *B. longum* 1714 was similar to a known immunoregulatory microbe that contains an immunomodulatory EPS and distinct from other bifidobacteria that do not express EPS by focusing on cytokine secretion and cell surface co-stimulatory or regulatory molecule expression. Subsequently, the influence of oral administration of *B. longum* 1714 on systemic aberrant inflammatory responses (assessed by measuring cytokines and the transcription factor NFκB), was determined when mice were challenged with LPS to induce systemic inflammation in a model of endotoxemia. Due to the possibility of *B. longum* 1714-induced changes in the microbiota effecting systemic *B. longum* 1714 effects on LPS challenge, these challenges were conducted in gnotobiotic Swiss Webster mice mono-colonized with *B. longum* 1714. While endotoxemia is an extreme model we also measured the systemic inflammatory responses in more physiologically relevant models, including obesity and stress. Finally, we examined the genome of this microbe and identified two potential mechanisms through which this strain could exert immunoregulatory effects – namely EPS production and tryptophan metabolism. These mechanisms were confirmed to exert effects on relevant immune cell models.

## Materials and methods

### Human immune cell co-cultures

Direct microscopic counts of freeze-dried bacterial cells were performed using a Petroff-Hausser counting chamber and PBS washed cells were co-incubated with peripheral blood mononuclear cells (PBMC) or monocyte-derived dendritic cells (MDDC). PBMCs were isolated from the peripheral blood of four healthy human donors and cultured in Dulbecco's Modified Eagle Medium- Glutamax TM (Gibco catalog 10,569–010), 10 % fetal bovine serum (Sigma catalog F4135), and 1 % penicillin/streptomycin (Sigma catalog P0781). PBMCs were incubated at a density of 2 × 10^5 cells per well for 24 h at 37 °C with 5 % CO2 in flat-bottomed 96-well plates. Each well received 20 μL of a bacterial suspension (with ratios of 100, 50, or 10 bacterial cells per PBMC) or isolated EPS, with doses ranging from 0.04 to 0.0025 mg. The incubation was conducted both with and without inflammatory stimuli, which included 100 ng/ml LPS-B5 Ultrapure (InvivoGen), 20 ng/ml recombinant human TNF-α (PeproTech), and 20 ng/ml recombinant human IFN-γ (PeproTech).

Human peripheral blood monocytes were isolated from PBMCs from six healthy human donors using MACS CD14 positive isolation (Miltenyi Biotec, 130–050–201). Cells were cultured in complete RPMI medium supplemented with 10 % fetal bovine serum, penicillin (100 U/ml), and streptomycin (0.1 mg/ml) (Sigma, Buchs, Switzerland) with IL-4 1000 U/ml (Novartis) and GM-CSF 1000 U/ml (PeproTech, 300–03) for 6 days to differentiate monocytes into MDDCs. MDDCs were incubated with individual bacterial strains (100, 10 bacterial cells to one MDDC) for 24 h. For cell surface staining, the following antibodies were used: PE-Cy7 anti-CD274, APC anti-CD273, Pacific blue anti-CD11c (eBioscience, Vienna, Austria), PE anti-DCIR, FITC anti-CD80 (BD Pharmingen), and AF 700 anti-CD86 (BD Pharmingen). Cell surface expression was evaluated by flow cytometry (Gallios system, Beckman Coulter, Nyon, Switzerland). Cytokine levels in the PBMC and MDDC culture supernatants were quantified and repeated in duplicate using the MILLIPLEX 42 Plex cytokine assay. NF-κB activation in THP-1 cells was quantified using a NF-κB-inducible SEAP reporter construct (InvivoGen).

### LPS challenge murine models

All animal experiments were performed in accordance with EU legislation (Directive 2010/63/EU) and ethical approval (B100/3866, B100/3716) was granted from the Animal Experimentation Ethics Committee of University College Cork.

Female BALB/c mice 6–8 weeks of age (Harlan UK) were housed in individually ventilated cages and provided *ad libitum* access to sterile standard mouse chow and water. Mice were randomized to receive PBS (*n* = 4) or 1714™ (*B. longum* NCIMB 41,676) (*B. longum* 1714) (*n* = 7) at a dose of 1 × 10^9^ CFU via oral gavage daily for 16 weeks. Additional animals administered PBS (*n* = 6) or *B. longum* 1714 (*n* = 10) were challenged with 1 mg/kg LPS (Sigma, L4391) via intraperitoneal (i.p.) injection. After 2 h, blood and spleens were isolated. Spleen single cell suspensions were stimulated for 48 h with anti-CD3 and anti-CD28 antibodies (BD Biosciences,Oxford, UK) or for 72 h with LPS (Sigma). The cytokine levels in the splenocytes culture supernatant and the mouse plasma was quantified using Meso Scale Discovery murine U-plex kits, and this was repeated in duplicate.

Female NFκB-lux transgenic mice on a C57BL/6J-CBA/J background (Charles River Laboratories, Wilmington, USA) were administered *B. longum* 1714 as a freeze-dried powder reconstituted in drinking water *ad libitum* at approximately 1 × 10^9^ CFU/day/animal, or a placebo control for 20 days prior to LPS i.p. challenge. *In vivo* imaging of NF-κB-lux transgenic mice were performed using the *In Vivo* Imaging System (IVIS, Xenogen, Alameda, USA) and imaged continuously for 5 min with a medium sensitivity setting starting 2 min after the injection of d-luciferin.

Gnotobiotic animals (female Swiss Webster mice) were administered a rifampicin resistant variant of *B. longum* 1714 at a dose of 1 × 10^7^ CFU/day (*n* = 12) or placebo (*n* = 6) for 14 days followed by 14-day washout period and challenged with 1 mg/kg LPS. Plasma was sampled 2 h post i.p. challenge with LPS and IL-1β measured using Meso Scale Discovery murine kit.

### Diet induced obesity murine model

Seven-week-old male C57BL/J6 mice were fed a low-fat diet (10 % calories from fat; Research Diets, New Jersey; #D12450B), a high-fat diet (DIO; 45 % calories from fat; Research Diets, New Jersey; #D12451) or a high-fat diet with *B. longum* 1714 (1 × 10^9^ CFU/day) in drinking water for 14 weeks. All mice were housed in groups of five and fresh *B. longum* 1714 aliquots were administered daily. Body weight and food intake were assessed weekly. After 14 weeks, isolated splenocytes were stimulated *in vitro* for 72 h with LPS and cytokines measured using Meso Scale Discovery murine U-plex kit.

### Stress induced neuroinflammatory murine model

Innately anxious male BALB/cOlaHsd (BALB/c) mice were administered *B. longum* 1714, or placebo for 6 weeks by oral gavage and challenged for the last 4 days with two separate acute stressors (tail suspension test and the forced swim test). Trunk blood was collected one hour post stressor and IL-1β levels in plasma was measured using an Meso Scale Discovery murine U-plex kit. The detailed protocol for this murine model has been described previously (Savignac et al., 2014).

### EPS isolation and characterization

MRS agar plates (3 % glucose) were used to grow *B. longum* 1714 for EPS extraction and purification. The isolation of EPS from *B. longum* 1714 and its characterization using monosaccharide analysis of size exclusion chromatography (SEC) fractions are identical to that previously described for *B. longum* 35624 (Altmann et al., 2016).

### Genome assessment

The genome of *B. longum* 1714 was sequenced and data assembled as described previously for *B. longum* 35624 (Altmann et al., 2016). The initial genome screen was based on the methodology devised in Valles-Colomer *et al*., 2019 which curated a module-based framework of microbial pathways from reference genomes of human gut isolates that would allow identification of modules involved in the degradation and biosynthesis of neurotransmitters, short chain fatty acids (SCFAs), proteases and amino acids. For further exploration of the tryptophan biosynthetic pathway in *B. longum* 1714, nucleotide sequences were retrieved from the KEGG pathway database Ortholog table (http://www.genome.jp/kegg). Amino acid sequences with homology to each tryptophan catalytic domain were obtained from an NCBI BLAST search of the *B. longum* NCC2705 database [BLAST x: NCBI database]. (http://www.ncbi.nlm.nih.gov/genomes/lproks.cgi). The Aromatic lactate dehydrogenase (aldh) genes were identified using NCBI BLASTn with default settings and a cutoff of 70 % identity and 70 % query coverage.

### Supernatant metabolomics

*B. longum* 1714 was cultured in distinct media to determine its tryptophan biosynthetic capacity (using low-salt mineral medium (LMM)) or tryptophan catabolism (using LMM or M9 minimal salts). Tryptophan production by *B. longum* 1714 from anthranilate and glucose was measured after 24 hour and 48-hour culture in LMM, while the production of tryptophan metabolites was measured after 3, 6, 16, 24 and 48-hours incubation in tryptophan-containing media (LMM and M9). Tryptophan and its metabolites were determined by HPLC-UV and LCMSMS. The metabolites quantified include anthranilate, indole-acrylic, hydroxy‑tryptophan, Indole-3-lactic acid, indole-3-aldehyde, Tryptamine, indole-3-acetic acid, indole-3-acetamide, kynurenine, serotonin, indole-3-propionic acid.

### Tryptophan plasma levels in humans

Healthy male and female participants, aged ≥18 to ≤45 years, with impaired sleep quality were randomized in a double-blind, placebo-controlled study, taking either a capsule containing *B. longum* 1714 at a dose of 1 × 10^9^ colony forming units (CFU), corn starch, and magnesium stearate or placebo containing corn starch, and magnesium stearate daily for 8 weeks, as described in detail in Patterson et al. (2024) . The Clinical Research Ethics Committee of the Cork Teaching Hospitals granted full approval to conduct the study on September 13, 2019 (AFCRO-108). The study was conducted according to the International Conference on Harmonization (ICH) Good Clinical Practice, in accordance with the most recent version of the Declaration of Helsinki on ethical principles for medical research in human subjects (ICH 1996). (ClinicalTrials.gov on 18/11/2019 under identifier NCT04167475.)”

Following protein and phospholipid removal, plasma samples were analysed using a Thermo Scientific Vanquish liquid chromatography coupled to Thermo Q Exactive hybrid quadrupole-Orbitrap mass spectrometer. Peak areas were extracted using Compound Discoverer 3.1 (Thermo Scientific). Metabolites that were detected in fewer than 10 % of samples were excluded from the analysis. Additional information can be found in supplementary Materials and Methods.

### Statistical analysis

All statistical analyses unless stated otherwise were performed using GraphPad Prism for Windows (Version 10.2). Data were examined for normality using the Kolmogorov – Smirnov test, and for homogeneity of variance. Statistical significance between groups with normal distribution was evaluated using a one-way analysis of variance (ANOVA) with Tukey post-tests, whereas data not normally distributed were analyzed using the Kruskal–Wallis’s test with Dunnett’s multiple comparison test. Principal component analysis (PCA) was performing using missMDA and FactoMineR, while ggbiplot was used to draw plots in R (Josse et al., 2012). Permutational multivariate analysis of variance (PERMANOVA) using the Adonis function in the R Vegan package was used to determine significance in dissimilarity matrices across samples by metadata categories (*e.g.*, cytokines, cell surface markers). Changes from baseline comparisons were analyzed using One-Way ANCOVA or Mann Whitney test. Unless otherwise indicated, all results are presented as means ± SEM. Differences were considered significant at *p < 0.05, **p < 0.01; ***p < 0.001.

## Results

### Strain-specific induction of PBMC cytokine secretion

**Fig. 1:**
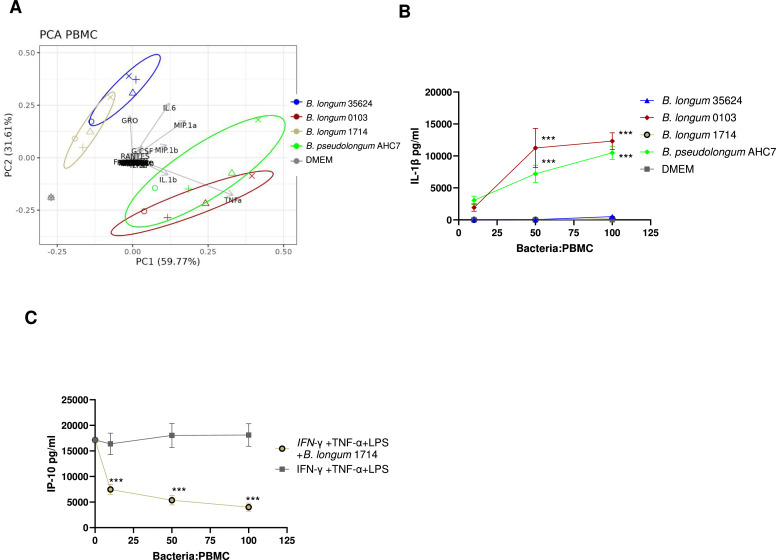
Peripheral blood mononuclear cells (PBMC) cytokine response to different bifidobacteria at different doses. PBMCs from four healthy donors were stimulated with different concentrations of bifidobacteria (100, 50, 10:1 PBMC) for 24 h and the cytokine levels in the culture supernatant were quantified and **(A)** Principal component analysis (PCA) comparison was performed of the cytokine response of PBMCs induced by all 4 tested strains at the 100:1 concentration*, B. longum* 1714 (light grey), *B. longum* 35624 (blue), *B. longum* 0103 (red) and *B. pseudolongum* AHC7 (green). **(B)** IL-1β level in the culture supernatant from the PBMC culture was quantified. Data are presented as line graphs with mean ± SEM values illustrated. **(C)** Inflammatory PBMC IP-10 response to *B. longum* 1714 at different doses. Inflammatory TNF-α, IFN-γ, and LPS stimulated PBMCs from three healthy donors were incubated with different concentrations of bifidobacteria (100, 50, 10:1 PBMC) for 24 h, and the IP-10 level in the culture supernatant was quantified. Data are presented as line graphs with mean ± SEM values illustrated. Statistical significance for the PBMC data was determined using the ANOVA and Dunnett’s multiple comparison ****p < 0.0001, ***p < 0.001 vs. media control.

Firstly, we wished to assess if the *in vitro* immune response to *B. longum* 1714 was similar but not identical to *B. longum* 35624 (a known immunoregulatory microbe that contains an immunomodulatory EPS), and distinct from other bifidobacteria that do not express EPS to the same extent. PBMCs were exposed to each bifidobacterial strain for 24 h, in the presence of antibiotics that kill each of these strains. Of the 42 cytokines measured, *B. longum* 1714, *B. longum* 35624, *B. longum* 0103 and *B. pseudolongum* AHC7 induced significant secretion of 12, 18, 23, or 30 cytokines respectively compared to unstimulated values, in a dose dependent manner (supplementary Table S1A). Principal component analysis of cytokine secretion from the stimulated PBMCs revealed that *B. longum* 1714 clustered closely with *B. longum* 35624 (PERMANOVA *p* = 0.065) but clustered separately from *B. pseudolongum* AHC7 (PERMANOVA *p* = 0.043) and *B. longum* 0103 (PERMANOVA *p* = 0.019). PBMC donors had a minor effect in the PCA contributing 7.7 % of the variance observed, while the bacterial strains had the dominant effect on cytokine secretion, explaining 78 % of the variance in PBMC cytokine secretion. The separation of the clusters was primarily driven by the higher levels of pro-inflammatory cytokines induced by *B. pseudolongum* AHC7 and *B. longum* 0103 (Fig. 1A). In addition, strain-to-strain comparisons confirmed these clusters as only 5 cytokines were significantly different between *B. longum* 1714 and *B. longum* 35624, whereas *B. longum* 0103 induced significantly higher levels of twenty-two cytokines and *B. pseudolongum* AHC7 induced significantly higher levels of thirty-six cytokines compared to *B. longum* 1714 (supplementary Table S1A). For example, *B. longum* 0103 and *B. pseudolongum* AHC7 induced significantly more IL-1β than *B. longum* 1714 and *B. longum* 35624 at multiple doses (Fig. 1B). Another pro-inflammatory cytokine induced by *B. longum* 0103 and *B. pseudolongum* AHC7, but not *B. longum* 1714 or *B. longum* 35624 was IP-10 (supplementary Fig. S1A). To test if intrinsic properties of the bacterial strain might actively inhibit IP-10 secretion, IP-10 secretion was induced by the inflammatory stimuli (IFN-γ, TNF-α and LPS). Pre-incubation of PBMCs with *B. longum* 1714 suppressed IP-10 secretion (Fig. 1C).

### Strain-specific induction of MDDC cytokine secretion, costimulatory molecule expression and tryptophan metabolism

**Fig. 2:**
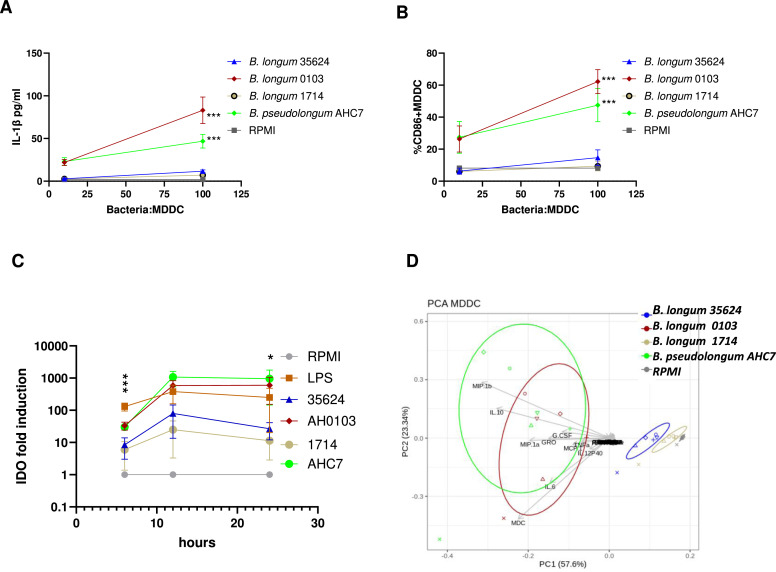
Monocyte derived dendritic cells (MDDC) immune response to different bifidobacteria at different doses. MDDC’s from six healthy donors were stimulated with different concentrations of bifidobacteria (100, 10:1 MDDC) for 24 h, the **(A)** IL-1β level of the culture supernatant and the **(B)** % of CD86+MDDCs was quantified. Data are presented as line graphs with mean ± SEM values illustrated. **(C)** Indoleamine 2,3-dioxygenase (IDO) gene expression in MDDCs response to different bifidobacteria or LPS at different time points. MDDC’s from three healthy donors were stimulated with bifidobacteria at 3,16, and 24 h. Results are shown as the mean +/- SE, *n* = 3 donors. Statistical significance was determined using the Kruskal-Wallis test and using the ANOVA and Dunnett’s multiple-comparison ****p < 0.0001, ***p < 0.001 vs. media control. **(D)** Principal component analysis (PCA). Comparison of the immune response of MDDC’s induced by all 4 tested strains, *B. longum* 1714 (light grey), *B. longum* 35624 (blue), *B. longum* 0103 (red) and *B. pseudolongum* AHC7 (green). The PCA clearly shows that the bacteria are all in separate clusters, indicating that the immune response induced is different between all these stimuli.

In addition to PBMCs, we investigated the MDDCs response to the same four bifidobacteria strains described above. Of the 42 cytokines measured, *B. longum* 1714, *B. longum* 35624, *B. longum* 0103 and *B. pseudolongum* AHC7 induced the secretion of 9, 14, 30, or 31 cytokines respectively (supplementary Table S1B). Similar to PBMCs, only *B. longum* 0103 and *B. pseudolongum* AHC7 induced secretion of IL-1β from MDDCs (Fig. 2A). Strain dependent effects were also observed on MDDC cell surface co-stimulatory and inhibitory molecule expression, especially for the costimulatory molecules CD80 and CD86 that were induced by *B. longum* 0103 and *B. pseudolongum* AHC7, but not *B. longum* 1714 or *B. longum* 35624 (supplementary Table S1C and Fig. 2B). Furthermore, the potential impact of bifidobacterial strains on MDDC tryptophan metabolism downstream of immune activation (Clarke et al., 2012) was assessed by quantifying indoleamine 2,3-dioxygenase (IDO) gene expression. IDO gene expression was enhanced significantly by LPS (positive control), *B. pseudolongum* AHC7 and *B. longum* 0103, while *B. longum* 35624 and *B. longum* 1714 induced low levels of expression (Fig. 2C). Principal component analysis of the MDDC cytokine and costimulatory molecule responses to these bifidobacteria revealed that *B. longum* 1714 clustered closely with *B. longum* 35624 (PERMANOVA *p* = 0.052) but clustered separately from *B. pseudolongum* AHC7 (PERMANOVA *p* = 0.004) and *B. longum* 0103 (PERMANOVA *p* = 0.003). Different donors only had a minor effect (1.3 %) on the MDDC results, while the different strains contributed to 57 % of the variance observed. The separation of the clusters was primarily driven by the induction of higher levels of pro-inflammatory cytokine secretion and cell surface molecule expression for *B. longum* AHC7 and *B. longum* 0103 (Fig. 2D). Strain-to-strain comparisons confirmed these clusters as only one cytokine difference was noted when comparing *B. longum* 1714 to *B. longum* 35624, whereas *B. longum* 0103 induced significantly higher levels of twenty-seven cytokines and four cell surface markers and *B. pseudolongum* AHC7 induced significantly higher levels of twenty-seven different cytokines and three cell surface markers compared to *B. longum* 1714 (supplementary Table S1B-C).

### *B. longum* 1714 induced immune regulatory responses in murine models

**Fig. 3:**
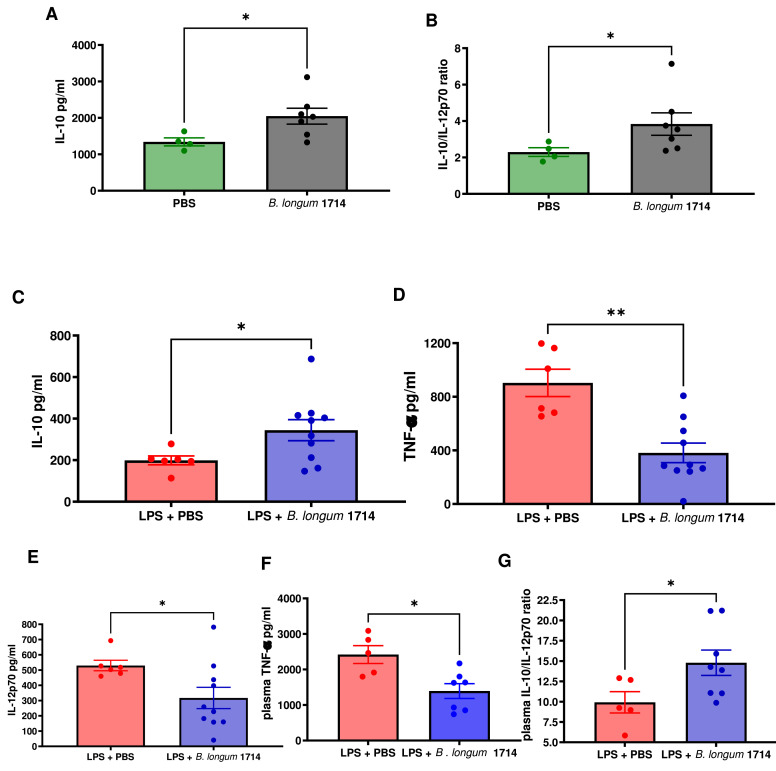
Peripheral cytokine response to *B. longum* 1714 in healthy and LPS exposed mice. **In healthy mice:** Splenocytes from both *B. longum* 1714 (*n* = 6) and PBS (*n* = 4) fed mice were isolated and stimulated with anti-CD3/CD28 for 48 h and the **(A)** IL-10 level **(B)** IL-10/IL-12p70 ratio of the culture supernatant was quantified. **In LPS exposed mice:** Splenocytes from *B. longum* 1714 (*n* = 10) and PBS (*n* = 6) fed mice were isolated 2 h following LPS administration and restimulated with either LPS for 72 h or anti CD3/CD28 for 48 h. **(C)** IL-10 levels were quantified in the LPS stimulated culture supernatant and **(D)** TNF-α and **(E)** IL-12p70 level were quantified for the anti CD3/CD28 stimulated culture supernatants. In addition, blood was extracted, and plasma isolated from these mice and the **(F)** TNF-α level and **(G)** IL-10/IL-12p70 ratio were quantified. Data are presented as scatter and bar plots with mean ± SEM values illustrated. Statistical significance was determined using the Mann Whitney test *p < 0.05; **p < 0.01 vs. PBS control.

As the PBMC and MDDC *in vitro* assays suggested that *B. longum* 1714 may possess some of the potent immunoregulatory features previously described for *B. longum* 35624, we investigated if *B. longum* 1714 could influence systemic immune responses following oral administration. BALB-C mice were orally gavaged *B. longum* 1714 or PBS control for 4 months and *ex vivo* splenocyte cytokine responses determined. Anti-CD3/CD28 stimulated splenocytes from *B. longum* 1714 exposed mice secreted significantly higher levels of IL-10 (Fig. 3A), associated with an increased IL-10/IL-12p70 ratio (Fig. 3B) compared to the PBS control. The immunomodulatory effects of *B. longum* 1714 was IL-10 specific, as no differences in splenocyte secretion of TNF-α, IFN-γ or IL-12p70 were observed (supplementary Fig. S1B-D).

**Fig. 4:**
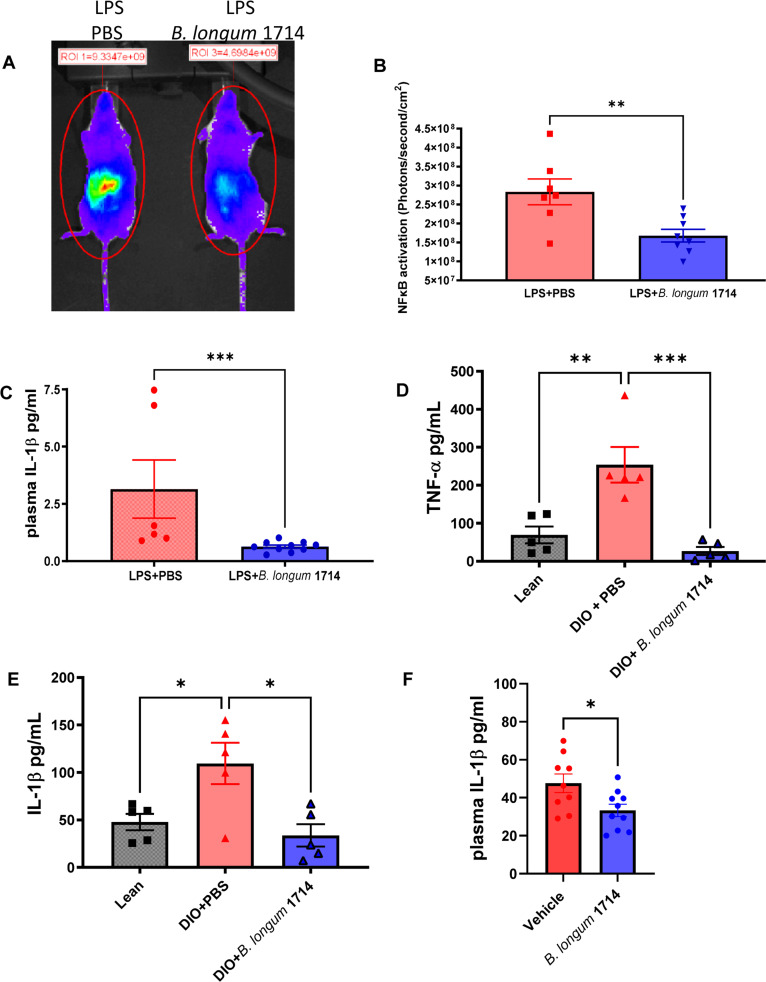
Peripheral inflammatory response to *B. longum* 1714 in LPS, high fat and stress exposed mice. **In LPS exposed mice: (A)** A representative *in vivo* image illustrating that whole body NFκB activation in NFκB-lux transgenic mice 1.5 h following LPS administration is attenuated when mice were pre-fed *B. longum* 1714. **(B)** The mean increase in NFκB activation (Photons/second/cm2) following i.p. injection of 0.5mg/kg of LPS is significantly less in isolated spleens 3 h post challenge in *B. longum* 1714 (*n* = 8) compared to PBS (*n* = 7) fed animals. Statistical significance was determined using the Mann Whitney test **p < 0.01; ***p < 0.001 vs. PBS control. Data are presented as scatter and bar plots with mean ± SEM values illustrated. **In LPS exposed gnotobiotic mice:** Blood was extracted, and plasma isolated from both *B. longum* 1714 (*n* = 10) and PBS (*n* = 6) fed mice 2 h post challenge with LPS 1mg/kg and the **(C)** IL-1β level was quantified. Statistical significance was determined using the Mann Whitney test *p < 0.01; ***p < 0.001 vs. PBS control. Data are presented as scatter and bar plots with mean ± SEM values illustrated. **In high fat exposed mice:** In diet induced obese mice (DIO) fed with *B. longum* 1714 (*n* = 5) and PBS (*n* = 5), and lean control mice, splenocytes were isolated and stimulated with LPS for 72 h and (**D)** TNF-α **(E)** and IL-1β levels were quantified in the culture supernatant. Data are presented as scatter and bar plots with mean ± SEM values illustrated. Statistical significance was determined using the Mann Whitney test *p < 0.05; **p < 0.01 vs. DIO PBS control. **In stress exposed mice:** Blood was extracted, and plasma isolated from both *B. longum* 1714 (*n* = 10), and PBS (*n* = 10) fed mice 1 hour’s post-acute stress and the **(F)** IL-1β level was quantified. Data for all graphs are presented as scatter and bar plots with mean ± SEM values illustrated. Statistical significance was determined between the change from baseline value using the Mann Whitney test *p < 0.05, *vs.* PBS control.

To determine if the *B. longum* 1714 induced increase in IL-10 secretion might be relevant for controlling aberrant inflammatory responses, mice were LPS challenged (i.p.) to induce systemic inflammation. Similar to non-challenged animals, splenocytes from *B. longum* 1714 exposed mice continued to secrete higher levels of IL-10, even after LPS challenge compared to the PBS control (Fig. 3C). In contrast, the increase in *ex vivo* anti-CD3/CD28 stimulated splenocyte secretion of TNF-α, (Fig. 3D) and IL-12p70 (Fig. 3E) in LPS challenged animals was significantly blunted in *B. longum* 1714 exposed animals. Similarly, plasma TNF-α levels were lower (Fig. 3F), associated with an increased plasma IL-10/IL-12p70 ratio (Fig. 3G) in *B. longum* 1714 exposed animals. As NF-κB is a key transcription factor for mediating the proinflammatory effects of LPS, we further explored if *B. longum* 1714 gavage could also impact *in vivo* NF-κB activation. Compared to placebo gavaged animals, pre-exposure of NFκBlux transgenic mice with *B. longum* 1714 decreased LPS-induced NF-κB activity *in vivo* (Fig. 4A) and in resected spleens isolated 3 h post LPS challenge (Fig. 4B). Finally, to assess if the *B. longum* 1714 protection against LPS challenge might be indirectly due to *B. longum* 1714-induced changes in the microbiota or if there was a direct effect on the immune system, we repeated the LPS challenge in gnotobiotic Swiss Webster mice mono-colonised with *B. longum* 1714. *B. longum* 1714 transited the gut at high levels during feeding and persisted, albeit at lower levels, up to two weeks after cessation of feeding (supplementary Fig. S1E). LPS-induced IL-1β levels in plasma were significantly lower in mice mono-colonised with *B. longum* 1714 than in mice that remained germ free, suggesting that *B. longum* 1714 directly influenced the immune response to LPS (Fig. 4C).

### *B. longum* 1714 reduces the increase in proinflammatory cytokines associated with obesity and stress induced inflammation

We next determined if *B. longum* 1714 might influence systemic inflammatory responses in more physiologically relevant models, including obesity and stress induced responses. Indeed, *B. longum* 1714 administration to C57BL/J6 obese mice fed a high fat diet resulted in reversal of the increased secretion of TNF-α (Fig. 4D) and IL-1β (Fig. 4E), by LPS-stimulated splenocytes, comparable to that seen with lean control animals.

In addition, innately anxious BALB/cOlaHsd (BALB/c) mice were used to study the effect of *B. longum* 1714 in influencing stress induced peripheral inflammation. *B. longum* 1714 significantly reduced plasma IL-1β in animals subjected to acute stress compared to the vehicle group (Fig. 4F).

### Characterization of *B. longum* 1714 associated EPS

**Fig. 5:**
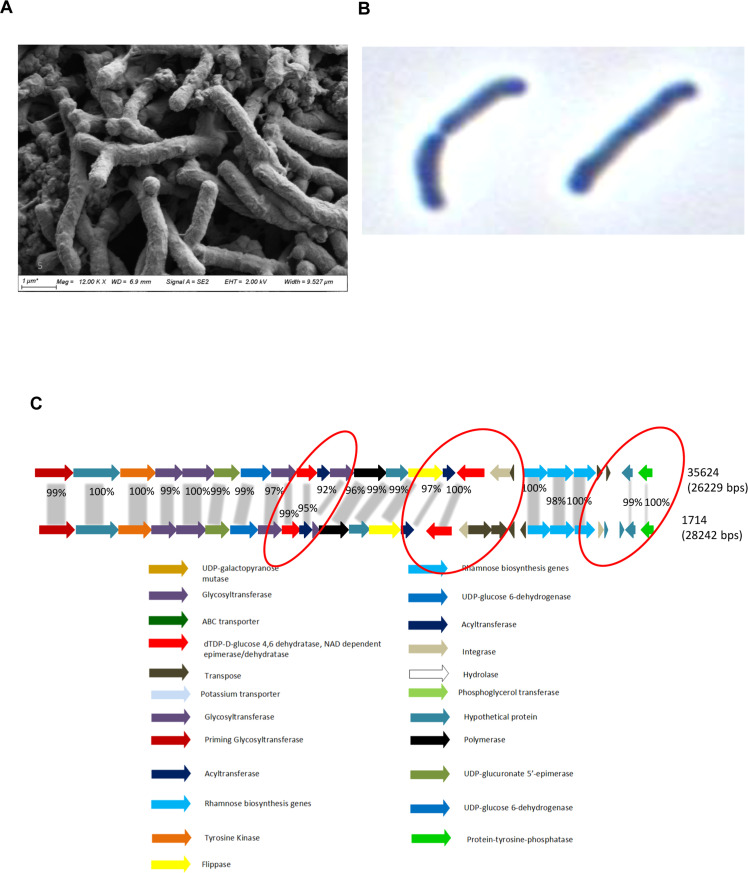
**(A)** Electron microscopy of *B. longum* 1714: morphology of *B. longum* 1714 and the layer of extracellular polysaccharide shown by scanning electron microscopy visible. Scale bars are indicated at the bottom right of each panel. **(B)** Microscopy of *B. longum* 1714: morphology of *B. longum* 1714 and the layer of extracellular polysaccharide shown by light microscopy when the strain is grown in milk. **(C)***B. longum* 1714 *versus B. longum* 35624 EPS Cluster 1. Illustration of the EPS cluster located in the *B. longum* 35624 genome and comparison to similar clusters located in *B. longum* 1714, Each gene is color-coded according to function which is indicated in the legend located at the end of the page. Percentages represent the percent of sequence similarity at the protein level with corresponding genes in the *B. longum* 35624 genomes. The locus tags of the first and last genes located in the EPS clusters of *B. longum* 1714 are also indicated in the illustration.

One potential mechanism for the immunomodulatory effects of *B. longum* 1714 could be mediated via its cell surface structures, such as EPS. Growth on Congo red agar plates showed that the colonies of *B. longum* 1714 and *B. longum* 35624 (EPS positive control) were convex, mucoid, and bright white, typical of an EPS-positive phenotype (supplementary Fig. S2A-B). In contrast, *B. longum* 0103 and *B. pseudolongum* AHC7 colonies (EPS negative controls) were pink/red indicating no EPS production (supplementary Fig. S2C-D). Electron and light microscopy confirmed the presence of an EPS layer on the cell surface of *B. longum* 1714 (Fig. 5A and B).

To better understand the *B. longum* 1714 EPS, a comparative analysis of the genome sequences, particularly those of extracellular structures, was performed between *B. longum* 1714 and *B. longum* 35624 (an EPS positive control). We identified a 26.2Kb gene cluster, predicted to encode the biosynthetic machinery for EPS biosynthesis in both strains. Comparison of this gene cluster showed two differences between the strains. *B. longum* 1714 lacks the second acetyltransferase present in *B. longum* 35624 (BI0357), and *B. longum* 1714 possesses a much shorter fifth glycosyltransferase (GT), including the priming GT, where a large part of the capsular polysaccharide synthesis protein is missing compared to *B. longum* 35624 (Fig. 5C). This truncated GT predicts a lower level of glucose in the *B. longum* 1714 associated EPS relative to *B. longum* 35624 associated EPS. The genes in the EPS cluster of *B. longum* 1714 and their predicted functions are fully described in Supplementary Table S2.

**Fig. 6:**
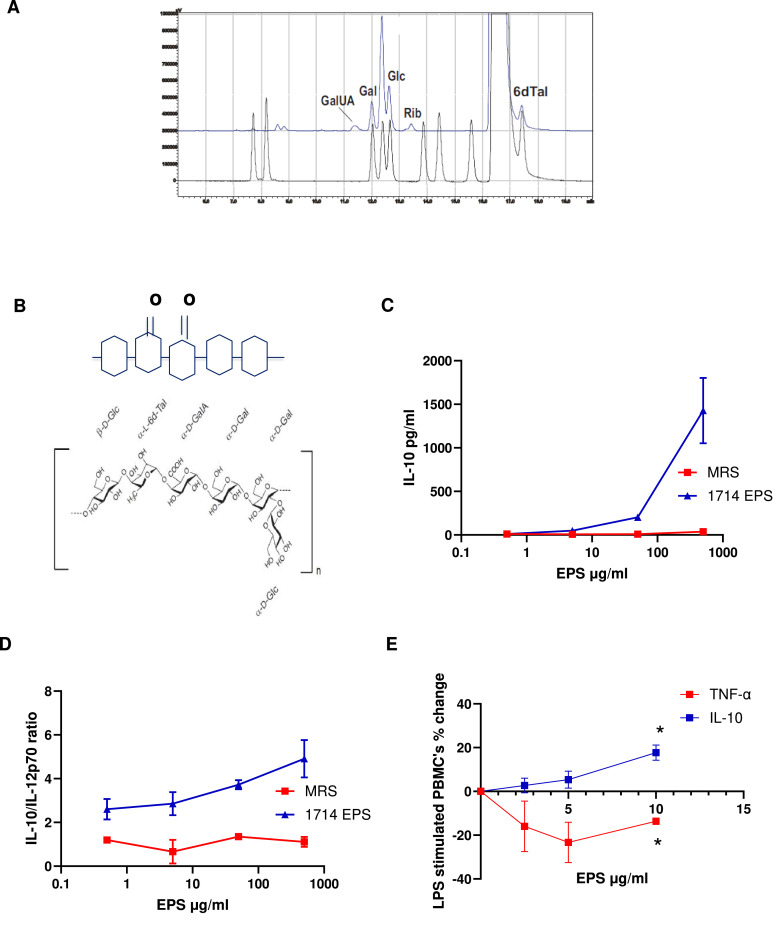
**(A)***B. longum* 1714 EPS characterization. HPLC analysis of anthranilic acid labeled monosaccharides of EPS revealed the presence of glucose (Glc), galactose (Gal), and 6-deoxy-talose (6dTal). **(B)***B. longum* 1714 EPS composition and structure. The structure is annotated as the chemical formula and in condensed form. PBMC cytokine response to EPS: PBMCs from 3 healthy donors were stimulated with different concentrations of crude EPS from *B. longum* 1714 for 24 h, and **(C)** IL-10 secretion into the culture supernatant and **(D)** IL-10/IL-12 ratio was quantified. **(E) D**ose dependent increase in IL-10 secretion and decrease in TNF-α section by EPS in LPS-stimulated PBMCs.

EPS purified from *B. longum* 1714 had an average mass greater than 1 MDa (Mw), as measured by high performance size exclusion chromatography (SEC), and was negatively charged, as measured by anion exchange. *B. longum* 1714 EPS was found to contain glucose (Glc), galactose (Gal), galacturonic acid (GalA) and 6-deoxy-talose (6dTal) (Fig. 6A).

The molar ratio of the constituent sugars Glc, Gal, GalA and a 6dTal approximated 2: 2: 1: 1 respectively.

This is in line with the *B. longum* 1714 EPS gene cluster composition prediction of repeating subunits that consists of five monosaccharides, of which one is an epimer of glucuronic acid, one or two others are either d-fucose or 6-deoxy-talose, some of which may be O-acetylated. Combining both gene cluster prediction and monosaccharide analysis data, the final predicted *B. longum* 1714 EPS composition consists of a branched hexasaccharide repeating unit containing two galactose and two glucose moieties, galacturonic acid and the sugar 6-deoxy-l-talose (Fig. 6B).

Importantly, when human PBMCs were stimulated with *B. longum* 1714-derived EPS, a dose dependent increase in IL-10 secretion (Fig. 6C) and IL-10/IL-12p70 ratio (Fig. 6D) was observed. In addition, *B. longum 1714*-derived EPS increased IL-10 secretion and reduced TNF-α secretion by LPS-stimulated PBMCs (Fig. 6E). The response to *B. longum* 1714-derived EPS is like that seen with the intact cell, suggesting that *B. longum* 1714 associated EPS might be relevant for some of its immunoregulatory effects.

### *B. longum* 1714 tryptophan synthesis and metabolism

In addition to the intrinsic cell wall associated immunoregulatory properties of *B. longum* 1714, we hypothesized that metabolites produced by this microbe might also influence the host. As previous studies suggest that *B. longum* 1714 consumption in humans may influence the gut-brain axis (Allen et al., 2016; Moloney et al., 2021; Patterson et al., 2024), we examined the genome of *B. longum* 1714 for genes known to be required for degradation or biosynthesis of neurotransmitters, SCFAs, proteases and amino acids. The *B. longum* 1714 genome contained genes involved in tryptophan synthesis and degradation, quinolinic acid degradation, glutamate synthesis, isovaleric acid synthesis II (KADC pathway), ClpB (ATP-dependent chaperone protein), S-Adenosylmethionine (SAM) synthesis, and acetate synthesis I (supplementary Table S3A). No *B. longum* 1714 gene modules were identified for γ-Aminobutyric acid (GABA), serotonin, kynurenine, tryptamine, and cortisol. We focused on the tryptophan biosynthesis pathway due to its relevance to immune and brain health. The genome of *B. longum* 1714 was further interrogated for the presence of tryptophan biosynthesis genes (supplementary Fig. S3). The genetic machinery for tryptophan biosynthesis is present in *B. longum* 1714 including anthranilate synthase subunit (trpE), which converts chorismate to anthranilate in the first committed step of tryptophan biosynthesis. Glutamine amidotransferase of anthranilate synthase (trpD_1) and anthranilate phosphoribosyltransferase (trpD_2) both convert anthranilate to N-(5′-phosphoribosyl)-anthranilate. Phosphoribosylanthranilate isomerase (PRAI), indole-3-glycerol phosphate synthase (IGPS), indoleglycerol phosphate aldolase (trpA), and tryptophan synthase (trpB) were also identified (supplementary Table 3B). In addition to genes for tryptophan biosynthesis we also looked for the genes for tryptophan metabolism (supplementary Fig. S4) and a BLAST search of the *B. longum* 1714 genome identified a homolog of an aromatic lactate dehydrogenase (99.7 % amino acid identity) previously found to be involved in the production of Indole-3-lactic acid (ILA) from tryptophan in a *B. longum* strain (Laursen et al., 2021).

**Fig. 7:**
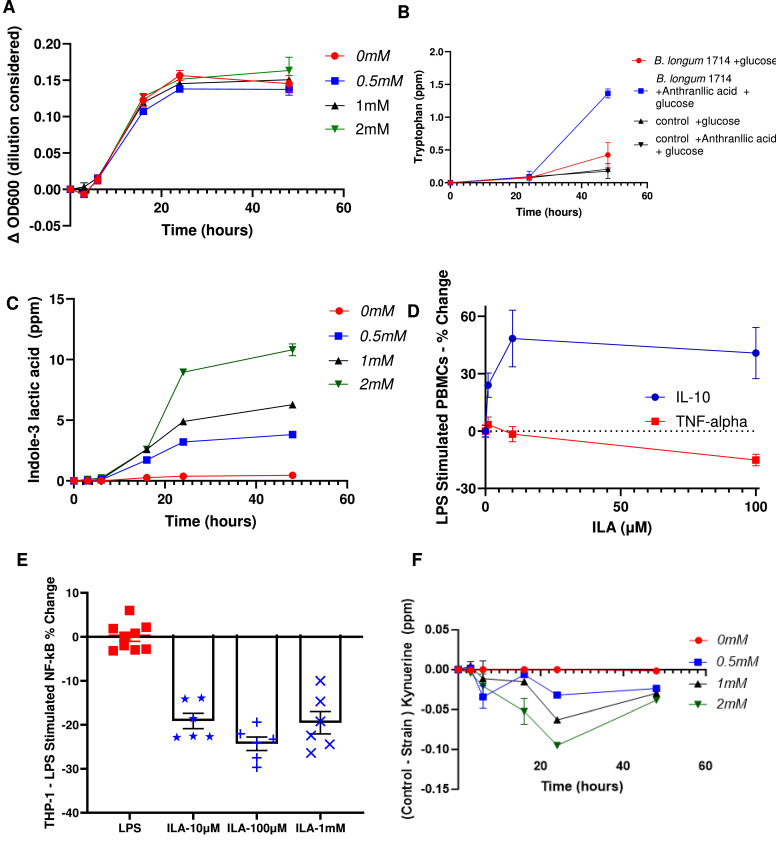
*In vitro* production of metabolites by *B. longum* 1714. **(A)** Growth of *B. longum* 1714 with different concentrations of tryptophan. **(B)***In vitro* production of tryptophan by *B. longum* 1714 with and without the tryptophan precursor anthranilic acid. **(C*)*** Dose dependent *in vitro* production of Indole-3-lactic acid (ILA) by *B. longum* 1714 with different concentrations of tryptophan. **(D)** Dose dependent increase in IL-10 secretion and decrease in TNF-α section by ILA in LPS-stimulated PBMCs **(E)** Dose dependent suppression of LPS-induced NF-κB activation in THP-1 cells by ILA **(F)** Dose dependent degradation of kynurenine during *B. longum* 1714 culture with different concentrations of tryptophan.

*B. longum* 1714 was able to grow in LMM media with or without tryptophan, which indicates the strain’s ability to synthesize tryptophan to support its growth as tryptophan is an essential amino acid for bacterial growth (Fig. 7A). Tryptophan precursor anthranilic acid levels significantly increased in *B. longum* 1714 cultures, peaking at 16 h before decreasing for the remainder of the 48-hour culture indicating anthranilic acid metabolism (supplementary Fig. S1F). In agreement, in *B. longum* 1714 cultures containing the anthranilic acid and glucose, tryptophan levels were significantly increased compared to glucose alone (Fig. 7B) Furthermore, ILA was produced by *B. longum* 1714 in a time and tryptophan concentration dependent manner (Fig. 7C). ILA increased IL-10 secretion and reduced TNF-α secretion by LPS-stimulated PBMCs (Fig. 7D), while also decreasing NF-κB activation in LPS-stimulated THP-1 cells (Fig. 7E). There was no detectable secretion of GABA, Indole-3-acetamide, Indole-acrylic, serotonin, Indole-3-propionic acid, or tryptamine, while kynurenine levels decreased in *B. longum* 1714 cultures, indicating that the strain was metabolizing kynurenine (Fig. 7F).

**Fig. 8:**
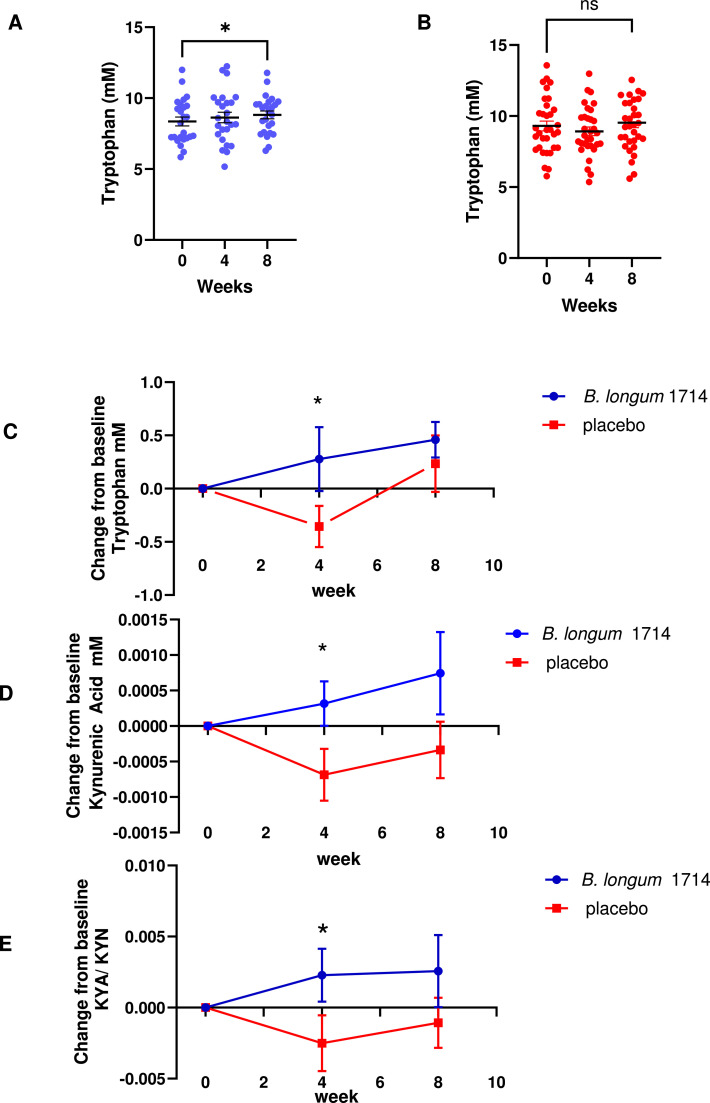
Plasma concentrations of tryptophan in **(A)***B. longum* 1714 (*n* = 25) and **(B)** placebo (*n* = 33) administered volunteers at baseline and following 4- or 8-weeks intervention. **(C)** Change from baseline levels of tryptophan, **(D)** change from baseline levels of kynurenic acid, **(E)** and change from baseline levels of kynurenic acid /L-kynurenine ratio were measured. Statistical significance was determined using ANOVA, Dunnett’s multiple comparison, *p < 0.05, *vs.* Week 0 and change from baseline value using the Mann Whitney test, *p < 0.05, *vs.* placebo .

Lastly, we quantified plasma tryptophan levels and associated metabolites in 58 healthy human subjects who were randomized to either placebo or *B. longum* 1714 consumption (1 × 10^9^ CFU) for 8 weeks (Patterson et al., 2024). In individuals consuming *B. longum* 1714, the mean plasma levels of tryptophan were 8.3 mM at baseline, 8.6 mM after 4 weeks treatment and significantly increased to 8.8 mM after 8 weeks treatment (Fig. 8A). Tryptophan levels did not significantly change in the placebo group over the same timeframe (Fig. 8B). When compared to placebo, the change from baseline levels of tryptophan were significantly increased at week 4 in those administered *B. longum* 1714 (Fig. 8C). For the downstream tryptophan metabolites, there was no difference in L-kynurenine concentrations observed between the two groups at any timepoint, but the neuroprotective kynurenic acid levels were significantly elevated in the *B. longum* 1714 group at week 8, which was not observed in the placebo group (supplementary Table S4) and there was a significant difference between *B. longum* 1714 and placebo group at week 4 when comparing their change from baseline levels (Fig. 8D). In addition, a comparison of the kynurenic acid:kynurenine ratio (broadly utilized as an indicator of neuroprotective: neurotoxic tryptophan metabolism) between the groups showed that 4 weeks of *B. longum* 1714 supplementation significantly increased this ratio compared to the placebo group (Fig. 8E). Finally, there was no significant differences in other tryptophan metabolites that we measured (supplementary Table S4).

## Discussion

In this study, we found that *B. longum* 1714 directly modulates the immune system by inducing high levels of the anti-inflammatory cytokine IL-10, while reducing secretion of proinflammatory cytokines such as IP-10 *in vitro*, and TNF-α and IL-1β in multiple murine models. Communication with the host immune system was mediated in part by intrinsic properties of the *B. longum* 1714 cell wall, its EPS, and by secretion of immunomodulatory tryptophan metabolites such as ILA.

*Bifidobacterium longum* is one of the first species shared between mother and baby early in life (Feehily et al., 2023) and their presence in the gut seems to be an important feature in centenarians (Kato et al., 2017). However, the immunomodulatory interactions of bifidobacteria are independent of phylogeny and strain-specific effects have been well described (Lyons et al., 2010; Nicola et al., 2016; Geva-Zatorsky et al., 2017). Distinct immune phenotypes and reaction intensities were induced by the four *Bifidobacterium* strains examined in this study. Strain-specific immunomodulation may be related to niche adaptation in different mammalian gut ecosystems and anatomical sites. *B. longum* 1714 and *B. longum* 35624 were originally isolated from intestinal tissue from elderly healthy individuals. It was hypothesized that probiotic candidates adhering to healthy human non-inflamed gastrointestinal mucosa would be capable of communicating effectively with the host immune system and potentially possess potent immunoregulatory properties (Konieczna et al., 2012a). In contrast, *B. longum* 0103 is a fecal isolate while *B. pseudolongum* AHC7 was isolated from canines. The immune response induced by *B. longum* 1714 and *B. longum* 35624 was more similar than that induced by the other strains and future studies will expand the range of *Bifidobacterium* strains to determine if human mucosal-associated bifidobacteria consistently induce more effective immunoregulatory responses compared to bifidobacteria from other sources.

The host response to infection is mediated by innate and acquired cellular and humoral immune reactions, designed to limit spread of the offending organism and to restore organ homeostasis. However, to limit the aggressiveness of collateral damage to host tissues, a range of regulatory constraints should be activated. Our studies show that *B. longum* 1714 modulates host innate TLR pro-inflammatory responses to microbial PAMPs. This included *in vitro* inflammatory assays and murine models where mice were LPS-challenged to induce systemic inflammation, obese mice with chronic systemic exposure to gut-derived MAMPs (Shoelson et al., 2007; Cani et al., 2008), and acutely stressed animals and humans whereby stress disrupts gut barrier function allowing LPS and other molecules to enter the bloodstream, leading to increased production of inflammatory cytokines (Kelly et al., 2016). Even without an established complex microbiota the *B. longum* 1714 strain alone protected against LPS challenge suggesting that this microbe directly influenced the immune system resulting in an altered response to LPS challenge. These results are not without limitations as the germ-free mice are hyperinflammatory at baseline. Future experiments should investigate monocolonization with other strains as we do not know in this model system how unique this response is to *B. longum* 1714. However, the aim of this experiment was not to compare with other strains but to determine if the *in vivo* effect could be replicated in the absence of a microbiota. Mechanisms underpinning microbial modulation of TLR responses can include the blockade of Iκ-B poly-ubiquination, the enhancement of NF-κB export from the nucleus, or the induction of regulatory cells and cytokines that dampen activation of TLR-activated cells (Zhou et al., 2015; O'Mahony et al., 2008; Riedel et al., 2006; Groeger et al., 2013; Collier-Hyams et al., 2002; Kelly et al., 2004).

Exopolysaccharides (EPS) are carbohydrate polymers expressed on the cell surface or secreted by bacteria for both protection and interaction with the surrounding environment (Castro-Bravo et al., 2018). EPS contributes to host–microbe interactions in multiple ways, including adhesion to the intestinal epithelium, protection from adverse environmental conditions and resistance to acid and bile stresses (Fanning et al., 2012; Alessandri et al., 2019; Ruas-Madiedo et al., 2010). Indeed, the successful transit and establishment of *B. longum* 1714 in gnotobiotic mice may be partly facilitated by its EPS. The significant inter- and intra-species variability in gene clusters responsible for EPS biosynthesis, structure, and composition, may also contribute to strain specific immune responses. The EPS from *B. longum* 1714 is a branched hexasaccharide repeating unit with two galactoses, one glucose, galacturonic acid and 6-deoxytalose, which has not been previously described. It is similar, but not identical, to the EPS from *B. longum* 35624 (Altmann et al., 2016). The EPS from *B. longum* 35624 induces IL-10 via TLR-2 and future studies are required to determine if the EPS from *B. longum* 1714 induces IL-10 via a similar mechanism (Schiavi et al., 2016; Schiavi et al., 2018; Wallimann et al., 2021). Regardless, this potent induction of IL-10 and reduction of pro-inflammatory TNF-α by EPS should contribute to the *in vivo* anti-inflammatory effects of *B. longum* 1714, as has been described for other microbes (Mackey and McFall, 2006; Chu and Mazmanian, 2013; Konieczna et al., 2012b).

Co-evolution of microbial communities within their hosts has resulted in intertwined metabolic pathways that affect physiological and pathological processes. Indeed, 64 % of human blood metabolites are significantly associated with either host genetics or the gut microbiome, with 69 % of these associations driven solely by the microbiome (Diener et al., 2022). Tryptophan derivatives of host and microbial origin are emblematic of this metabolic promiscuity (Agus et al., 2018; Gheorghe et al., 2019; Zelante et al., 2021). *B. longum* 1714 contains genes for tryptophan biosynthesis enzymes (Valles-Colomer et al., 2019; Schell et al., 2002), which were previously described also for *Escherichia coli* and bifidobacteria (Zhao et al., 2011). *B. longum* 1714 supplementation increased plasma tryptophan levels, however the biological impact of increasing tryptophan levels by 0.5 mM is unclear. Tryptophan plasma levels are known to be influenced by dietary intake and activity of the three metabolic pathways (kynurenine, 5-hydroxytryptamine, and indole pathways). Of particular importance is the activity level of the interferon-induced indoleamine-2,3-dioxygenase (IDO) pathway, which can deplete tryptophan levels as was clearly observed in COVID-19 patients (Almulla et al., 2022). Most dietary tryptophan is used by the host for protein synthesis while microbial derived tryptophan is more relevant for microbial production of indoles within the gut and potentially could be available for mucosal immune signaling pathways, although this remains to be experimentally demonstrated. In fact, lower levels of tryptophan production and tryptophan metabolism by gut microbes may increase the risk of severe and chronic outcomes to SARS-CoV-2 infection due to impaired innate and adaptive responses to infection (Yao et al., 2024). The direct contribution of microbial-derived tryptophan to plasma tryptophan levels is highly unlikely to be substantial, and future experimental models will need to be performed to untangle the effects of diet, microbial production, and host metabolism on regulating peripheral tryptophan levels. In addition, plasma concentrations may not accurately reflect gut lumen concentrations or microbial tryptophan consumption and future experiments examining tryptophan levels in faecal samples will be required to better determine the importance of microbial tryptophan generation on gut lumen concentrations.

In addition to tryptophan biosynthesis, *Bifidobacterium longum* strains can metabolize tryptophan into indole-related compounds such as indoleacetate (IAA), indolepropionate (IPA) and indole-3-lactate (ILA) (Smith and Macfarlane, 1996; Russell et al., 2013; Laursen et al., 2021). *B. longum* 1714 expresses the aromatic lactate dehydrogenase (ALDH) gene previously shown to be required for ILA generation (Laursen et al., 2021), and ILA production by *B. longum* 1714 in culture supernatants was confirmed by mass spectrometry. Importantly, we also showed that ILA enhanced IL-10 secretion and suppressed TNF-α secretion and NF-κB activation in LPS-stimulated cells. ILA signals via the AhR and hydroxycarboxylic acid receptor 3 (HCA3) with significant effects already reported on epithelial barrier function, immune cell activation and neuronal differentiation (Ehrlich et al., 2020; Meng et al., 2020; Roager and Licht, 2018; Laursen et al., 2021; Wong et al., 2020).

The immunoregulatory effects of *Bifidobacterium longum* were shown using *in vitro* experiments measuring cytokines, cell surface costimulatory and regulatory molecule expression, and in murine models measuring cytokines and the activity of the transcription factor NF-κB. However, a deeper analysis of immune cell phenotypes and transcriptional responses will provide further insights into the immunological mechanisms underpinning the protective effects of this strain.

In conclusion, our results show that *B. longum* 1714 has significant effects on the innate immune system and these effects may be mediated partially via cell surface associated macromolecules and secreted immunomodulatory and neuroprotective metabolites. However other potential mechanisms, currently unknown, may also exert effects of the strain on host immune responses.

## Disclosures

Competing Interests Statement: 35624™ and 1714™ are registered trademarks of Novozymes A/S and the cultures are in commercial products.

## Funding

The work in part was supported by grants from Novozymes and Proctor and Gamble. Some of the authors are supported by a Science Foundation Ireland research center grant 12/RC/2273_P2 and a Science Foundation Ireland Frontiers for the Future Award 21/FFP-A/10000. Some of the authors are also funded by the Health Research Board (HRB) through Health Research Awards (grants no HRA_POR/2011/23; TGD, and GC, HRA_POR/2012/32; TGD and HRA-POR-2–14–647: GC, TGD) and through EU GRANT 613979(MYNEWGUT FP7-KBBE-2013–7: TGD).

## Credit author statement

**David Groeger:** Writing-Original Draft, Supervision, Investigation, Conceptualization, Formal analysis, **Lu Yao:** Investigation, **Fergus Collins:** Investigation, **Ida Søgaard Larsen:** Formal analysis **Hern-Tze Tina Tan:** Project administration, Investigation, **Selena Healy:** Investigation, Formal analysis. **Valentina Ambrogi:** Methodology, Investigation **Karolina Tykwinska:** Supervision, **Martin Schmidt:** Methodology, Investigation **Patrick Golletz:** Investigation, **Barry Kiely:** Supervision, Funding acquisition, Conceptualization **Gerard Clarke:** Supervision, Funding acquisition, Conceptualization **Timothy G. Dinan:** Supervision, Funding acquisition, Conceptualization **Eileen F. Murphy:** Investigation, Conceptualization, **Liam O’Mahony:** Writing-Original Draft, Supervision, Funding acquisition, Conceptualization, **All authors** contributed to Review & Editing the paper and all authors agreed the final version for submission.

## Declaration of competing interest

The authors declare the following financial interests/personal relationships which may be considered as potential competing interests:

David Groeger reports financial support was provided by Novonesis. Fergus Collins reports financial support was provided by Novonesis. Ida Sogaard Larsen reports financial support was provided by Novonesis. Hern-Tze Tina Tan reports financial support was provided by Novonesis. Selena Healy reports financial support was provided by Novonesis. Valentina Ambrogi reports financial support was provided by Novonesis. Karolina Tykwinska reports financial support was provided by Novonesis. Martin Schmidt reports financial support was provided by Novonesis. Patrick Golletz reports financial support was provided by Novonesis. Barry Kiely reports financial support was provided by Novonesis. Eileen F. Murphy reports financial support was provided by Novonesis. Liam O’Mahony reports financial support was provided by The Procter and Gamble Company. Liam O’Mahony reports a relationship with Novonesis that includes: consulting or advisory. Liam O’Mahony has received research grants from GSK, Chiesi and Fonterra, and participated in speaker bureau for Nestle, Yakult, Reckitt and Abbott. Gerard Clarke has received honoraria from Janssen, Probi, and Apsen as an invited speaker; is in receipt of research funding from Pharmavite, Fonterra, Reckitt, Nestle and Tate and Lyle; and is a paid consultant for Yakult, Zentiva and Heel Pharmaceuticals. This support neither influenced nor constrained the contents of this paper. If there are other authors, they declare that they have no known competing financial interests or personal relationships that could have appeared to influence the work reported in this paper.

## Appendix Supplementary materials

Image, application 1

Image, application 2

Image, application 3

Image, application 4

Image, application 5

Image, application 6

Image, application 7

Image, application 8

Image, application 9
