# Supplementary material for: Complementary immunoregulatory effects of Bifidobacterium longum 1714TM associated exopolysaccharide and tryptophan metabolism
Source: Curr Res Microb Sci. 2025 Sep 28;9:100481. doi: 10.1016/j.crmicr.2025.100481 (PMC12546897; doi:10.1016/j.crmicr.2025.100481)
Supplement: Supplementary file 1 [file mmc1.pdf]

Supplementary Figure S1

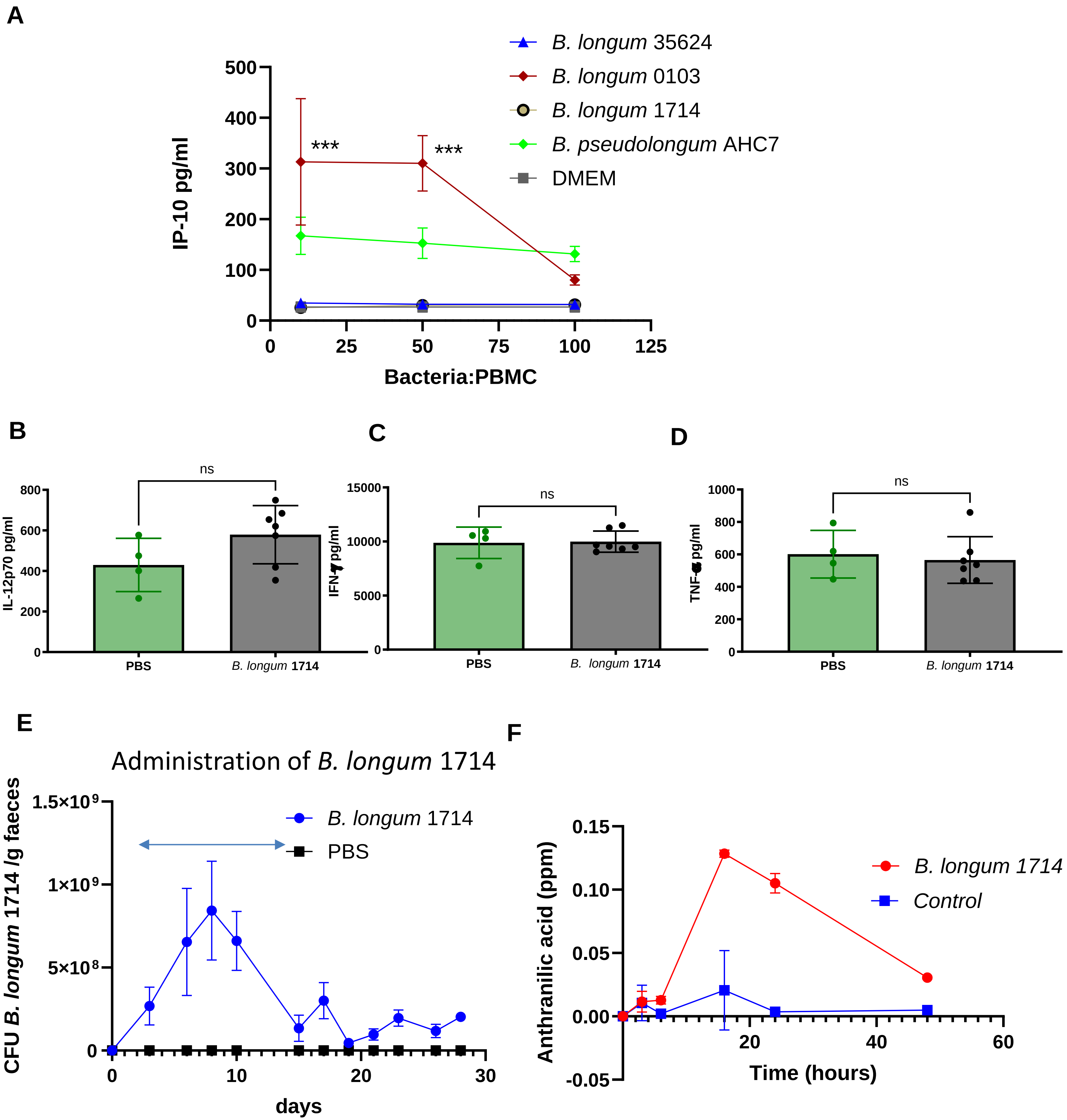

Supplementary Fig. S1**(A)**. Peripheral blood mononuclear cells (PBMC) cytokine response to different bifidobacteria at different doses. PBMCs from four healthy donors were stimulated with different concentrations of bifidobacteria (100, 50, 10:1 PBMC) for 24 h and the cytokine levels in the culture supernatant was quantified and IP-10 level in the culture supernatant from the PBMC culture was quantified. Data are presented as line graphs as mean ± SEM values illustrated. Statistical significance for the PBMC data was determined using the ANOVA and Dunnett's multiple comparison ****$p < 0.0001$, ***$p < 0.001$ vs. media control. Supplementary Fig. 1. Peripheral cytokine response to B. longum 1714 in healthy mice. Splenocytes from both B. longum 1714 (n=6) and PBS (n=4) fed mice were isolated and stimulated with anti-CD3/CD28 for 48 hours and the **(B)** IL-12p70, **(C)** IFN-γ, **(D)** TNF-α level of the culture supernatant was quantified. Data are presented as scatter and bar plots with mean ± SEM values illustrated. Statistical significance was determined using the Mann Whitney test vs. PBS control. Supplementary Fig. 1**(E)**. The differential transit of *B. longum* 1714 levels in the placebo (n=6) or *B. longum* 1714 (n=12) fed gnotobiotic mice after 14 days consumption and 14-day washout. Supplementary Fig. 1**(F)**. *In vitro* production of anthranilic acid by *B. longum* 1714.
