# Supplementary material for: Complementary immunoregulatory effects of Bifidobacterium longum 1714TM associated exopolysaccharide and tryptophan metabolism
Source: Curr Res Microb Sci. 2025 Sep 28;9:100481. doi: 10.1016/j.crmicr.2025.100481 (PMC12546897; doi:10.1016/j.crmicr.2025.100481)
Supplement: Supplementary file 2 [file mmc2.pdf]

Supplementary Figure S2

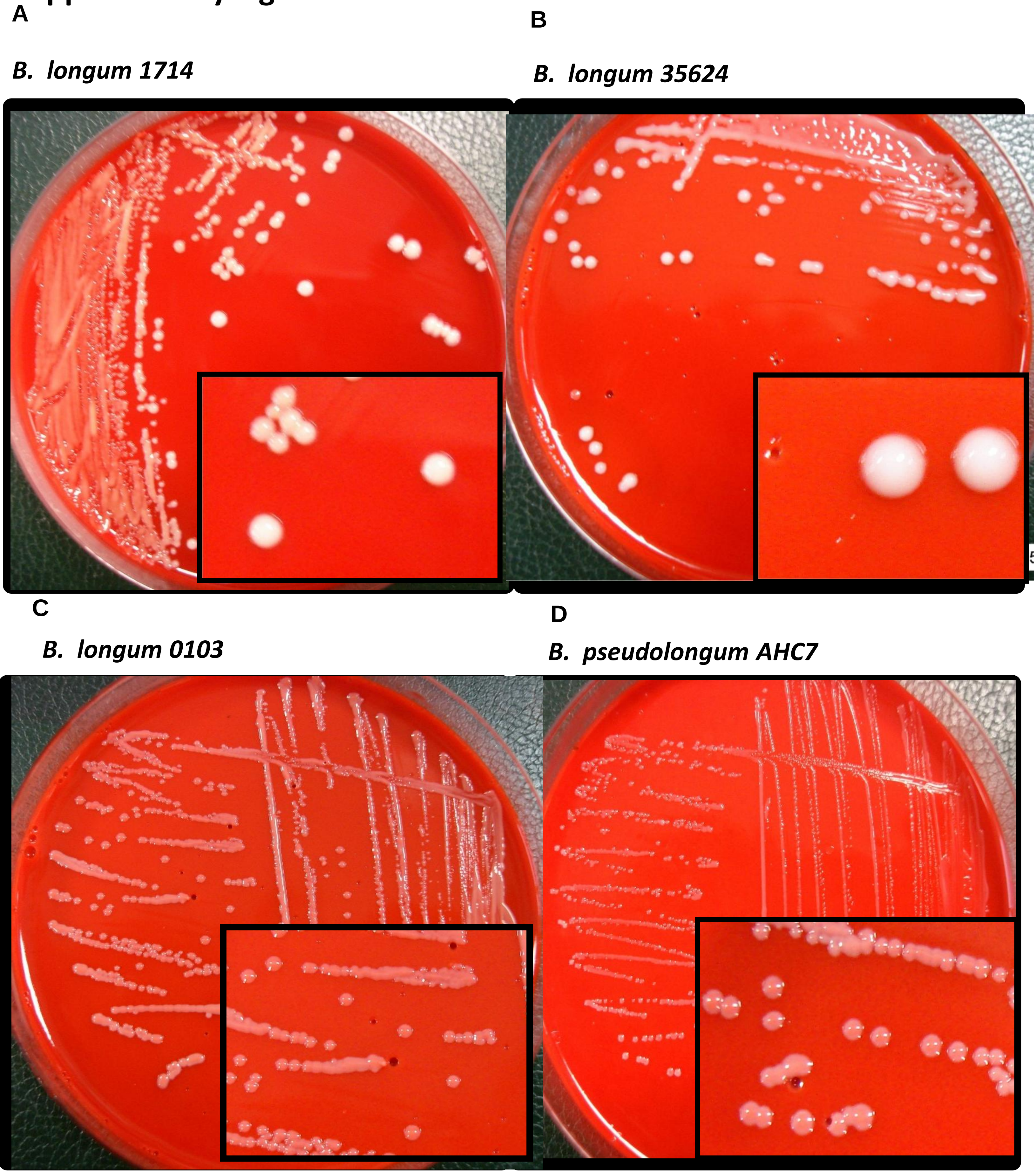

Supplementary Fig. S2. **(A-D)** EPS production by different bifidobacteria incubated at 37° C for 48-72 hours on Congo Red Agar.
