# Supplementary figures and images for: Complementary immunoregulatory effects of Bifidobacterium longum 1714TM associated exopolysaccharide and tryptophan metabolism
Source: Curr Res Microb Sci. 2025 Sep 28;9:100481. doi: 10.1016/j.crmicr.2025.100481 (PMC12546897; doi:10.1016/j.crmicr.2025.100481)

## Supplementary Figure S3

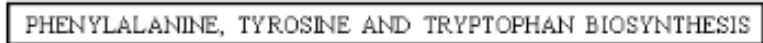

Supplementary Fig. S3. KEGG pathway for tryptophan biosynthesis

Supplement: Supplementary file 3 [file mmc3.pdf]

## Supplementary Figure S4

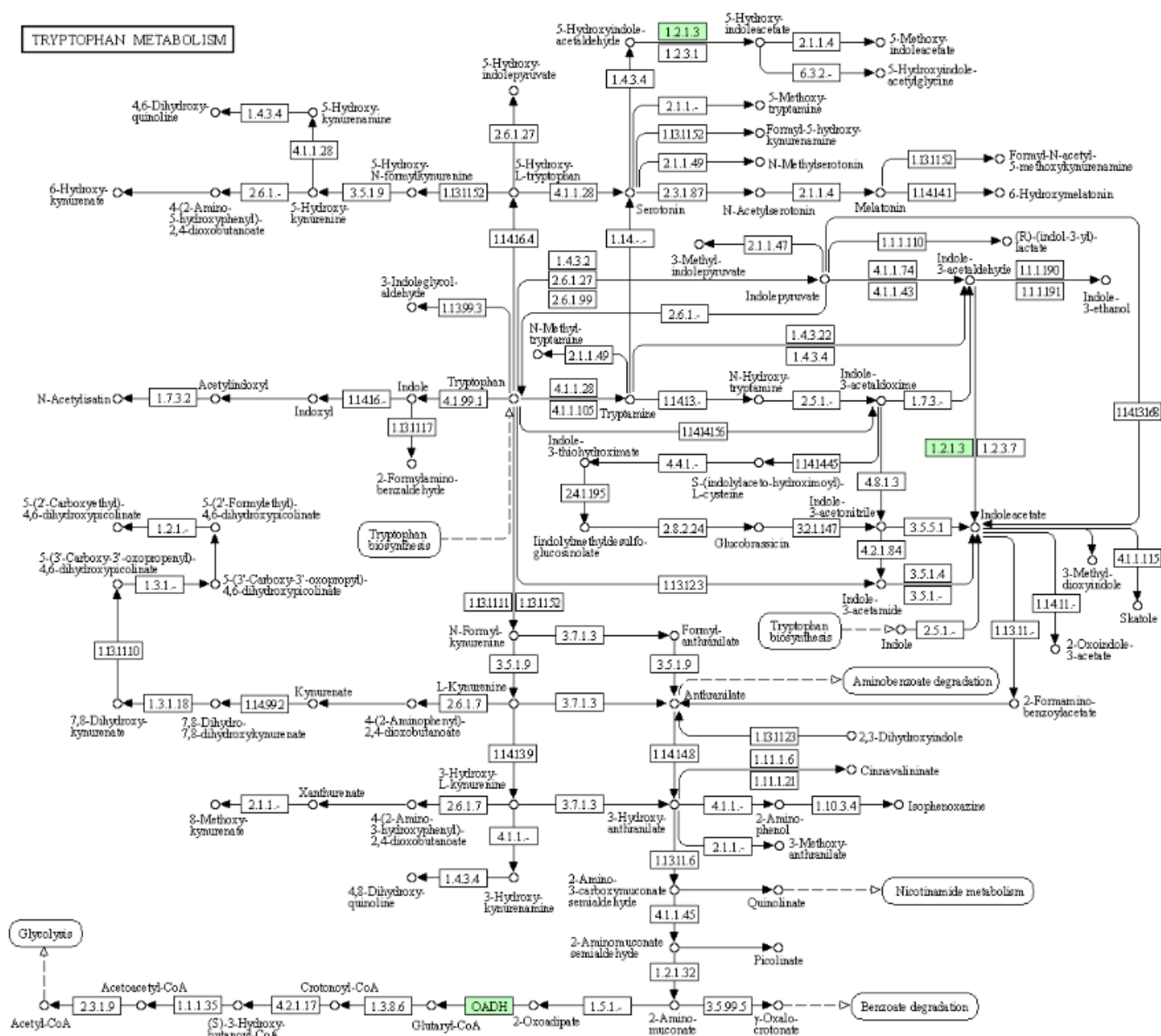

Supplementary Fig. S4. KEGG pathway for tryptophan metabolism

Supplement: Supplementary file 4 [file mmc4.pdf]
