# Supplementary material for: Complementary immunoregulatory effects of Bifidobacterium longum 1714TM associated exopolysaccharide and tryptophan metabolism
Source: Curr Res Microb Sci. 2025 Sep 28;9:100481. doi: 10.1016/j.crmicr.2025.100481 (PMC12546897; doi:10.1016/j.crmicr.2025.100481)
Supplement: Supplementary file 5 [file mmc5.pdf]

Supplementary

MATERIALS AND METHODS

In vivo assessment of B. longum 1714 transit in a germ-free model.

Rifampicin resistant variant of *B. longum* 1714 was administered to female Swiss Webster mice (gnotobiotic, 10-12 weeks of age) for 14 days followed by 14-day washout period. The trial consisted of two groups, PBS (n=6), and *B. longum* 1714 (n=12) via oral gavage at a dose of 1 X 10^7 CFU/day. The mice were bred in-house and maintained in sterile flexible film isolators. Mice were housed in individually ventilated cages in the Germfree Unit and fed sterilized standard pellet diet (Envigo, Cambridgeshire, UK) and water *ad libitum*. All food, water bedding and other supplies needed for the mice in the isolator were sterilized by autoclaving. Standard housing and environmental conditions were maintained (temperature 21°C, 12 hours light, and 12 hours darkness with 50% humidity) in the animal housing facility. Transit levels of *B. longum* 1714 were monitored in fecal pellets during feeding on day 1, 3, 7, 10, and during washout period on day 15, 17, 19, 21, 23, 26 and 28. Detection of *B. longum* 1714 was facilitated by isolating a spontaneous rifampicin resistant variant of the strain. Incorporation of rifampicin in the TPY plates was used to assess transit to ensure that only the fed rifampicin resistant bifiobacteria was cultured. Faecal samples were collected daily and *B. longum* transit through the gastrointestinal tract was confirmed with specific PCR primers.

EPS production on Congo red agar

Congo red agar plates were used to phenotypically screen for exopolysaccharide (EPS) expressing bacterial strains. *B. longum* 1714, *B. longum* 35624® (positive control), *B. longum* 0103 and *B. pseudolongum* AHC7 (negative controls) were grown twice in reinforced clostridial medium (RCM) broth containing 0.05% cysteine and 5% sucrose for 48 hours at 37°C anaerobically. Cultures were streaked onto Skim Milk Congo Red agar containing 0.5% sucrose and 0.25% Congo Red and incubated for 48 hours at 37°C anaerobically. EPS production was assessed by using the inoculation loop “String test” and by evaluating colony appearance.

Analysis of Gamma-aminobutyric acid in vitro

Prior to analysis of Gamma-aminobutyric acid (GABA) concentration, the *Bifidobacterium longum* 1714 was sub-cultured in RCM containing 0.05% L-cysteine and grown anaerobically at 37°C for 72 hour under 3 different conditions; RCM alone, supplemented with 10 mg/ml of monosodium glutamate (MSG) and 40 mg/ml of MSG, each with their own negative control in which the strain was absent. One ml of the culture broth was centrifuged at 14,000 rpm for 1 min and the levels of GABA in the culture supernatant were determined with a Beckmann 6300 High Performance Amino Acid Analyser.

Scanning electron microscopy

Freeze-dried cells of the B. longum 1714 strain were initially resuspended in 30% ethanol for 24 hours followed by incubation in an increasing gradient of ethanol concentrations (40%, 50%, 60%, 70%, 80%, and 90%) for 24 hours at each step. Cells were then resuspended in 50% hexamethyldisilazane (Merck) for 12 hours, followed by 100% hexamethyldisilazane for an additional 12 hours. Cells were fixed to a stub and coated in gold using an Emitech K575X sputter-coater (Quorum Technologies, UK). Cells were then imaged on a Gemini field emission scanning electron microscope (ZEISS, Germany) at an accelerating voltage of 2kV.
