# Supplementary material for: Complementary immunoregulatory effects of Bifidobacterium longum 1714TM associated exopolysaccharide and tryptophan metabolism
Source: Curr Res Microb Sci. 2025 Sep 28;9:100481. doi: 10.1016/j.crmicr.2025.100481 (PMC12546897; doi:10.1016/j.crmicr.2025.100481)
Supplement: Supplementary file 6 [file mmc6.pdf]

Supplementary Table S1 (A). PBMC cytokine secretion in response to Bifidobacteria. Statistical significance for the PBMC data was determined using the ANOVA and Dunnett's multiple comparison ****p < 0.0001, ***p < 0.001,**p < 0.01, ,*p < 0.05, compared to media control or Compared to 1714.

		DMEM	35624100:1	35624 50:1	35624 10:1	0103 100:1	0103 50:1	0103 10:1	1714 100:1	1714 50:1	1714 10:1	AHC7 100:1	AHC7 50:1	AHC7 10:1
**IL-10**	Mean	5.964	842.3	61.51	5.809	475.4	2582	740.4	433.5	8.237	5.601	3312	5156	1827
	Std. Deviation	6.578	88.57	9.376	3.43	204.4	1020	373.6	138.1	3.381	3.81	2143	1158	563.2
	Std. Error of Mean	3.289	44.29	4.688	1.715	102.2	509.9	186.8	69.06	1.691	1.905	1071	579.1	281.6
	Compared to media control		ns	ns	ns	ns	***	ns	ns	ns	ns	****	****	*
	Compared to 1714.		ns	ns	ns	ns	***	ns	N/A	N/A	N/A	****	****	*
**TNF-α**	Mean	11.81	3860	425.1	24.31	32631	36707	14321	1351	36.13	10.34	30727	28884	19327
	Std. Deviation	1.781	1406	232.1	12.24	7628	7293	7804	183.3	17.4	5.504	6353	9680	6723
	Std. Error of Mean	0.8906	702.8	116	6.122	3814	3647	3902	91.67	8.698	2.752	3177	4840	3362
	Compared to media control		ns	ns	ns	****	****	**	ns	ns	ns	****	****	****
	Compared to 1714.		ns	ns	ns	****	****	**	N/A	N/A	N/A	****	****	****
**EGF**	Mean	43.83	80	50.83	41.01	59.17	66.29	64.94	67.39	40.13	57.05	60.45	93.22	82.45
	Std. Deviation	11.21	25.35	9.316	7.772	6.575	4.286	13.03	19.44	10.77	15.9	6.353	37.24	16.71
	Std. Error of Mean	5.606	12.67	4.658	3.886	3.287	2.143	6.516	9.718	5.383	7.949	3.176	18.62	8.354
	Compared to media control		*	ns	ns	ns	ns	ns	ns	ns	ns	ns	**	*
	Compared to 1714.		ns	ns	ns	ns	ns	ns	N/A	N/A	N/A	ns	***	ns
**Eotaxin**	Mean	29.02	122.9	107.1	53.71	104.8	108.6	110.7	114.1	60.55	23.14	116.3	133.1	136.2
	Std. Deviation	21.09	51.72	48.04	17.96	36.39	44.46	51.52	55.37	12.96	17.31	46.07	83.08	86.83

		DMEM	35624100:1	35624 50:1	35624 10:1	0103 100:1	0103 50:1	0103 10:1	1714 100:1	1714 50:1	1714 10:1	AHC7 100:1	AHC7 50:1	AHC7 10:1
	Std. Error of Mean	10.54	25.86	24.02	8.979	18.2	22.23	25.76	27.68	6.482	8.654	23.04	41.54	43.42
	Compared to media control		ns	ns	ns	ns	ns	ns	ns	ns	ns	ns	*	*
	Compared to 1714.		ns	ns	ns	ns	ns	ns	N/A	N/A	N/A	ns	ns	*
**FGF-2**	Mean	10.88	84.19	74.86	25.88	77.47	73.32	81.06	81.92	34.59	20.18	81.66	111.3	105
	Std. Deviation	7.691	24.48	11.52	12.33	12.13	10.15	13.6	31.01	12.11	8.888	15.03	71.35	61.73
	Std. Error of Mean	3.845	12.24	5.762	6.165	6.064	5.074	6.801	15.5	6.057	4.444	7.517	35.68	30.86
	Compared to media control		*	*	ns	*	*	*	*	ns	ns	*	***	***
	Compared to 1714.		ns	ns	ns	ns	ns	ns	N/A	N/A	N/A	ns	**	**
**FLT-3L**	Mean	12.21	21.51	21.84	11.96	18.74	26	20.31	29.24	11.78	7.857	26.31	34.06	31.58
	Std. Deviation	5.199	2.182	11.56	4.678	5.72	1.456	8.234	13.91	2.242	6.649	2.76	15.57	6.918
	Std. Error of Mean	2.6	1.091	5.782	2.339	2.86	0.7278	4.117	6.957	1.121	3.324	1.38	7.786	3.459
	Compared to media control		ns	ns	ns	ns	ns	ns	*	ns	ns	ns	**	*
	Compared to 1714.		ns	ns	ns	ns	ns	ns	N/A	N/A	N/A	ns	**	**
**Fractalkine**	Mean	70.97	573.2	420.4	131.6	382.2	610.6	472.3	578.2	169	109	624.6	738.3	728.3
	Std. Deviation	47.05	166.2	114.6	34.96	53.54	115	92.83	195.9	39.88	16.87	58.13	304.4	152.2
	Std. Error of Mean	23.52	83.09	57.29	17.48	26.77	57.5	46.41	97.94	19.94	8.437	29.06	152.2	76.11
	Compared to media control		****	**	ns	*	****	**	****	ns	ns	****	****	****
	Compared to 1714.		ns	ns	ns	ns	***	**	N/A	N/A	N/A	ns	****	****

		DMEM	35624100:1	35624 50:1	35624 10:1	0103 100:1	0103 50:1	0103 10:1	1714 100:1	1714 50:1	1714 10:1	AHC7 100:1	AHC7 50:1	AHC7 10:1
**G-CSF**	Mean	23.97	5238	338.2	38.43	2276	1999	655.6	2081	55.17	20.33	3773	4548	3163
	Std. Deviation	7.696	3027	164.8	15.18	1681	571.9	161.9	779.9	10.22	7.957	2245	1985	1443
	Std. Error of Mean	3.848	1513	82.4	7.592	840.4	285.9	80.94	389.9	5.111	3.979	1123	992.4	721.3
	Compared to media control		****	ns	ns	ns	ns	ns	ns	ns	ns	**	***	*
	Compared to 1714.		*	ns	ns	ns	ns	ns	N/A	N/A	N/A	ns	***	*
**GM-CSF**	Mean	5.117	246.9	32.36	7.12	932.2	282.6	70.34	94.98	10.75	3.769	429	233.6	165.5
	Std. Deviation	4.162	64.42	7.415	3.681	363.8	36.44	23.75	32.57	4.776	2.407	113.6	70.78	58.35
	Std. Error of Mean	2.081	32.21	3.708	1.84	181.9	18.22	11.88	16.28	2.388	1.204	56.78	35.39	29.18
	Compared to media control		*	ns	ns	****	*	ns	ns	ns	ns	****	ns	ns
	Compared to 1714.		ns	ns	ns	****	*	ns	N/A	N/A	N/A	**	ns	ns
**GRO**	Mean	152.1	18164	9996	591	4584	7036	10158	16341	1532	227.6	6241	10655	14430
	Std. Deviation	55.07	3948	1420	325.5	1068	455	1578	4832	334.6	75.25	1493	886.5	1744
	Std. Error of Mean	27.54	1974	709.9	162.8	533.9	227.5	789.1	2416	167.3	37.62	746.3	443.2	871.9
	Compared to media control		****	****	ns	*	***	****	****	ns	ns	**	****	****
	Compared to 1714.		ns	****	ns	****	**	****	N/A	N/A	N/A	****	****	****
**IL-1α**	Mean	5.297	96.58	9.836	0.863	1939	1890	267.1	33.36	0.7527	0.08139	1872	1466	648.6
	Std. Deviation	10.56	37.85	5.214	1.061	519.8	866.9	145.5	14.78	0.9251	0.1628	441.5	581.4	327.3
	Std. Error of Mean	5.278	18.93	2.607	0.5304	259.9	433.5	72.73	7.389	0.4625	0.08139	220.8	290.7	163.6

		DMEM	35624100:1	35624 50:1	35624 10:1	0103 100:1	0103 50:1	0103 10:1	1714 100:1	1714 50:1	1714 10:1	AHC7 100:1	AHC7 50:1	AHC7 10:1
	Compared to media control		ns	ns	ns	****	****	ns	ns	ns	ns	****	****	ns
	Compared to 1714.		ns	ns	ns	****	****	ns	N/A	N/A	N/A	****	****	ns
**IL-1RA**	Mean	21.19	196.4	94.46	35.59	134.2	270.9	195	134.3	38.93	25.98	295.4	412.8	266.1
	Std. Deviation	9.409	34.49	29.09	7.935	29.35	28.25	58.25	18.52	13.26	19.32	94.26	51.62	34.32
	Std. Error of Mean	4.704	17.24	14.54	3.967	14.68	14.12	29.13	9.262	6.629	9.658	47.13	25.81	17.16
	Compared to media control		****	ns	ns	**	****	****	**	ns	ns	****	****	****
	Compared to 1714.		ns	ns	ns	ns	****	****	N/A	N/A	N/A	****	****	****
**IL-2RA**	Mean	7.121	37.55	24.91	5.836	23.31	38.2	32.26	31.21	11.33	4.494	44.85	47.99	46.79
	Std. Deviation	6.941	10.06	6.262	4.779	8.171	9.47	5.762	3.302	12.33	7.463	9.997	19.14	31.67
	Std. Error of Mean	3.471	5.03	3.131	2.39	4.085	4.735	2.881	1.651	6.167	3.731	4.998	9.568	15.83
	Compared to media control		*	ns	ns	ns	*	ns	ns	ns	ns	**	***	***
	Compared to 1714.		ns	ns	ns	ns	*	*	N/A	N/A	N/A	ns	**	**
**IL-3**	Mean	1.839	1.217	0.443	0.2721	0.7712	0.407	0.5048	0.8666	0.2066	0.498	0.5394	3.527	0.426
	Std. Deviation	3.284	1.617	0.66	0.441	1.036	0.814	0.6837	1.62	0.3432	0.982	0.9233	4.905	0.4394
	Std. Error of Mean	1.642	0.8084	0.33	0.2205	0.5179	0.407	0.3419	0.81	0.1716	0.491	0.4617	2.452	0.2197
	Compared to media control		ns	ns	ns	ns	ns	ns	ns	ns	ns	ns	ns	ns
	Compared to 1714.		ns	ns	ns	ns	ns	ns	N/A	N/A	N/A	ns	ns	ns
**IL-4**	Mean	1.783	18.99	10.3	1.44	15.9	16.03	14.64	16.06	3.74	1.899	30.09	30.01	23.55

		DMEM	35624100:1	35624 50:1	35624 10:1	0103 100:1	0103 50:1	0103 10:1	1714 100:1	1714 50:1	1714 10:1	AHC7 100:1	AHC7 50:1	AHC7 10:1
	Std. Deviation	1.29	6.415	4.725	1.499	4.007	3.641	6.601	2.759	3.264	2.472	14.55	5.595	6.866
	Std. Error of Mean	0.6449	3.208	2.362	0.7495	2.004	1.82	3.3	1.379	1.632	1.236	7.277	2.797	3.433
	Compared to media control		**	ns	ns	*	*	*	*	ns	ns	****	****	****
	Compared to 1714.		ns	ns	ns	ns	*	*	N/A	N/A	N/A	*	****	****
**IL-5**	Mean	1.944	1.428	0.6034	0.4175	1.278	1.064	0.618	0.8978	0.1189	0.1053	1.75	3.961	1.988
	Std. Deviation	3.564	0.4416	0.5149	0.4303	0.8574	0.5128	0.5376	0.2046	0.2378	0.08958	0.7058	5.249	0.8359
	Std. Error of Mean	1.782	0.2208	0.2575	0.2151	0.4287	0.2564	0.2688	0.1023	0.1189	0.04479	0.3529	2.624	0.4179
	Compared to media control		ns	ns	ns	ns	ns	ns	ns	ns	ns	ns	ns	ns
	Compared to 1714.		ns	ns	ns	ns	ns	ns	N/A	N/A	N/A	ns	*	ns
**IL-6**	Mean	11.34	22166	2055	14.37	8074	18181	5973	10671	36.62	6.379	15836	29963	30849
	Std. Deviation	7.248	10232	881.5	7.473	5217	13138	1976	5312	17.93	3.484	12249	29477	34436
	Std. Error of Mean	3.624	5116	440.7	3.736	2609	6569	988.1	2656	8.963	1.742	6125	14738	17218
	Compared to media control		ns	ns	ns	ns	ns	ns	ns	ns	ns	ns	*	*
	Compared to 1714.		ns	ns	ns	ns	ns	ns	N/A	N/A	N/A	ns	*	*
**IL-7**	Mean	25.23	148.5	108.1	40.44	136.6	153.7	134.6	142.6	57.44	26.28	162.3	192.2	187.8
	Std. Deviation	7.946	38.76	31.28	15.87	21.62	43.01	44.44	36.28	6.68	7.691	24.78	76.93	74.54
	Std. Error of Mean	3.973	19.38	15.64	7.934	10.81	21.5	22.22	18.14	3.34	3.845	12.39	38.47	37.27
	Compared to media control		***	*	ns	**	***	**	**	ns	ns	***	****	****

		DMEM	35624100:1	35624 50:1	35624 10:1	0103 100:1	0103 50:1	0103 10:1	1714 100:1	1714 50:1	1714 10:1	AHC7 100:1	AHC7 50:1	AHC7 10:1
	Compared to 1714.		ns	ns	*	ns	*	**	N/A	N/A	N/A	ns	***	***
**IL-9**	Mean	2.346	2.895	1.786	0.2597	1.572	2.148	1.962	1.619	0.476	0.2417	2.586	6.312	3.371
	Std. Deviation	4.125	1.575	0.4926	0.2784	0.2594	0.387	0.392	0.3004	0.469	0.2855	0.2984	7.477	1.479
	Std. Error of Mean	2.062	0.7874	0.2463	0.1392	0.1297	0.1935	0.196	0.1502	0.2345	0.1427	0.1492	3.738	0.7395
	Compared to media control		ns	ns	ns	ns	ns	ns	ns	ns	ns	ns	ns	ns
	Compared to 1714.		ns	ns	ns	ns	ns	ns	N/A	N/A	N/A	ns	*	ns
**IL-12p40**	Mean	6.073	334.1	51.58	5.45	937	3843	1221	123.1	13.25	2.982	2142	2819	1912
	Std. Deviation	2.543	170.2	14.2	3.113	600.9	1819	957.9	31.94	10.08	5.964	1387	1607	736.1
	Std. Error of Mean	1.271	85.12	7.101	1.556	300.4	909.5	479	15.97	5.041	2.982	693.7	803.7	368
	Compared to media control		ns	ns	ns	ns	****	ns	ns	ns	ns	*	***	*
	Compared to 1714.		ns	ns	ns	ns	****	ns	N/A	N/A	N/A	*	***	*
**IL-12p70**	Mean	3.721	22.17	9.917	1.73	379.8	2492	545.8	16.37	2.98	1.06	507.8	482.9	309.1
	Std. Deviation	5.237	6.308	1.004	1.701	199.7	1702	451.3	5.841	1.728	1.002	336.4	359.9	136.7
	Std. Error of Mean	2.619	3.154	0.5019	0.8506	99.83	850.9	225.6	2.921	0.8642	0.501	168.2	180	68.36
	Compared to media control		ns	ns	ns	ns	****	ns	ns	ns	ns	ns	ns	ns
	Compared to 1714.		ns	ns	ns	ns	ns	ns	N/A	N/A	N/A	ns	ns	ns
**IL-13**	Mean	6.47	17.74	10.46	4.122	15.85	21.62	20.01	17.23	5.949	1.874	27.22	30.06	24.5
	Std. Deviation	5.528	1.881	2.541	2.572	3.543	3.035	3.829	4.363	3.077	2.109	7.769	10.68	6.301
	Std. Error of Mean	2.764	0.9406	1.271	1.286	1.772	1.517	1.914	2.182	1.538	1.054	3.884	5.338	3.151

		DMEM	35624100:1	35624 50:1	35624 10:1	0103 100:1	0103 50:1	0103 10:1	1714 100:1	1714 50:1	1714 10:1	AHC7 100:1	AHC7 50:1	AHC7 10:1
	Compared to media control		*	ns	ns	ns	**	**	*	ns	ns	****	****	***
	Compared to 1714.		ns	ns	ns	ns	***	***	N/A	N/A	N/A	ns	****	****
**IL-15**	Mean	2.363	7.074	2.374	2.236	3.372	4.659	5.795	4.231	1.061	0.8061	6.347	7.567	5.108
	Std. Deviation	4.384	3.541	0.8307	0.8731	1.626	1.07	1.691	3.284	0.416	0.4908	1.165	3.825	4.048
	Std. Error of Mean	2.192	1.77	0.4154	0.4365	0.8129	0.535	0.8454	1.642	0.208	0.2454	0.5824	1.913	2.024
	Compared to media control		ns	ns	ns	ns	ns	ns	ns	ns	ns	ns	ns	ns
	Compared to 1714.		ns	ns	ns	ns	ns	ns	N/A	N/A	N/A	ns	**	ns
**IL-17**	Mean	3.02	11.73	6.013	1.086	5.654	7.422	7.596	12.43	1.675	0.9109	7.106	12.93	10.91
	Std. Deviation	4.609	3.32	1.376	0.9656	0.4281	1.676	1.769	3.672	1.267	0.8028	0.9854	9.299	4.121
	Std. Error of Mean	2.304	1.66	0.6881	0.4828	0.2141	0.838	0.8845	1.836	0.6336	0.4014	0.4927	4.649	2.061
	Compared to media control		*	ns	ns	ns	ns	ns	**	ns	ns	ns	**	*
	Compared to 1714.		ns	ns	ns	ns	ns	ns	N/A	N/A	N/A	ns	***	**
**IFN-α**	Mean	8.15	91.75	47.78	18.31	90.6	125	103.2	71.22	19.28	5.853	118.2	126.3	112.5
	Std. Deviation	4.033	21.8	10.56	3.732	10.48	14.78	34.64	17.53	9.069	2.688	13.2	42.52	32.24
	Std. Error of Mean	2.016	10.9	5.281	1.866	5.239	7.391	17.32	8.767	4.535	1.344	6.602	21.26	16.12
	Compared to media control		****	ns	ns	****	****	****	**	ns	ns	****	****	****
	Compared to 1714.		ns	ns	ns	ns	ns	ns	N/A	N/A	N/A	ns	***	**
**IFN-γ**	Mean	7.02	43.02	19.49	8.125	485.8	2682	620.1	35.19	8.133	2.79	1676	839	476.1

		DMEM	35624100:1	35624 50:1	35624 10:1	0103 100:1	0103 50:1	0103 10:1	1714 100:1	1714 50:1	1714 10:1	AHC7 100:1	AHC7 50:1	AHC7 10:1
	Std. Deviation	8.619	11.22	2.09	2.214	125.2	650.7	545.4	17.06	1.685	1.264	889	540.3	224.9
	Std. Error of Mean	4.31	5.612	1.045	1.107	62.61	325.4	272.7	8.53	0.8424	0.6322	444.5	270.2	112.4
	Compared to media control		ns	ns	ns	ns	****	ns	ns	ns	ns	****	*	ns
	Compared to 1714.		ns	ns	ns	ns	***	ns	N/A	N/A	N/A	****	*	ns
**IP-10**	Mean	26.7	31.93	32.27	34.85	80.13	310.2	312.9	31.33	30.15	25.7	131.6	152.9	167.2
	Std. Deviation	20.55	18.05	7.442	15.35	20.15	109.5	249.7	16.18	14.38	12.61	30.73	60.39	73.41
	Std. Error of Mean	10.28	9.027	3.721	7.673	10.07	54.76	124.9	8.091	7.19	6.305	15.36	30.19	36.71
	Compared to media control		ns	ns	ns	ns	***	***	ns	ns	ns	ns	ns	ns
	Compared to 1714.		ns	ns	ns	ns	***	***	N/A	N/A	N/A	ns	ns	ns
**MCP-1**	Mean	1835	29161	25446	3693	841.1	7938	30255	29579	6948	2019	4184	16847	29972
	Std. Deviation	1992	9974	12084	3315	380.3	5106	10426	10273	3452	1778	3082	6501	7127
	Std. Error of Mean	996.1	4987	6042	1657	190.1	2553	5213	5137	1726	888.8	1541	3251	3563
	Compared to media control		****	***	ns	ns	ns	****	****	ns	ns	ns	*	****
	Compared to 1714.		ns	**	ns	****	ns	****	N/A	N/A	N/A	****	ns	****
**MCP-3**	Mean	16.62	212.9	230.9	38.11	47.76	90.59	721.4	252.9	70.64	35.37	55.9	136.4	339
	Std. Deviation	22.44	61.59	110.9	34.05	11.59	41.76	530.4	123.9	23.04	22.03	9.899	13.98	111.8
	Std. Error of Mean	11.22	30.79	55.45	17.02	5.795	20.88	265.2	61.97	11.52	11.01	4.95	6.99	55.9
	Compared to media control		ns	ns	ns	ns	ns	****	ns	ns	ns	ns	ns	ns

		DMEM	35624100:1	35624 50:1	35624 10:1	0103 100:1	0103 50:1	0103 10:1	1714 100:1	1714 50:1	1714 10:1	AHC7 100:1	AHC7 50:1	AHC7 10:1
	Compared to 1714.		ns	ns	ns	ns	ns	ns	N/A	N/A	N/A	ns	ns	ns
**MDC**	Mean	47.11	141.4	94.17	38.42	67.83	72.07	69.94	87.67	49.04	25.07	65.75	80.16	79.29
	Std. Deviation	26.66	79.62	44.34	19.54	23.88	29.52	32.82	28.07	12.2	18.75	16.3	37.77	24.42
	Std. Error of Mean	13.33	39.81	22.17	9.772	11.94	14.76	16.41	14.04	6.102	9.374	8.15	18.88	12.21
	Compared to media control		**	ns	ns	ns	ns	ns	ns	ns	ns	ns	ns	ns
	Compared to 1714.		ns	ns	ns	ns	ns	ns	ns	N/A	N/A	ns	ns	ns
**MIP-1α**	Mean	15.24	20945	2666	34.76	15371	20710	12544	13588	74.87	15.47	21138	27829	29961
	Std. Deviation	6.858	6218	1060	10.7	10495	9152	8173	5648	24.66	12.47	7036	11459	20189
	Std. Error of Mean	3.429	3109	530	5.35	5247	4576	4086	2824	12.33	6.237	3518	5729	10095
	Compared to media control		*	ns	ns	ns	*	ns	ns	ns	ns	**	***	***
	Compared to 1714.		ns	ns	ns	ns	*	ns	N/A	N/A	N/A	ns	***	***
**MIP-1β**	Mean	79.92	6941	2566	217.4	7323	11850	7096	4786	376.1	107	13518	14472	9521
	Std. Deviation	14.07	2010	681.6	74.09	3620	6020	2964	1613	103.8	23.02	8312	7211	3605
	Std. Error of Mean	7.037	1005	340.8	37.05	1810	3010	1482	806.6	51.89	11.51	4156	3605	1803
	Compared to media control		ns	ns	ns	ns	**	ns	ns	ns	ns	***	****	*
	Compared to 1714.		ns	ns	ns	ns	**	ns	N/A	N/A	N/A	*	***	*
**PDGF-AA**	Mean	115	165.9	128.3	111.3	111.1	123.9	127.2	134.2	117.4	123.9	121.4	173.6	171.6
	Std. Deviation	29.91	60.74	42.4	32.64	33.24	48.61	46.21	57.83	47.57	51.69	46.4	34.54	44.45
	Std. Error of Mean	14.95	30.37	21.2	16.32	16.62	24.31	23.11	28.92	23.79	25.84	23.2	17.27	22.23

		DMEM	35624100:1	35624 50:1	35624 10:1	0103 100:1	0103 50:1	0103 10:1	1714 100:1	1714 50:1	1714 10:1	AHC7 100:1	AHC7 50:1	AHC7 10:1
	Compared to media control		ns	ns	ns	ns	ns	ns	ns	ns	ns	ns	ns	ns
	Compared to 1714.		ns	ns	ns	ns	ns	ns	N/A	N/A	N/A	ns	ns	ns
**PDGF-BB**	Mean	0	11.55	0	0	0	0.4698	0	0	0	1.417	0.6502	30.4	23.36
	Std. Deviation	0	23.11	0	0	0	0.9396	0	0	0	2.834	1.3	44.5	32.21
	Std. Error of Mean	0	11.55	0	0	0	0.4698	0	0	0	1.417	0.6502	22.25	16.1
	Compared to media control		ns	ns	ns	ns	ns	ns	ns	ns	ns	ns	ns	ns
	Compared to 1714.		ns	ns	ns	ns	ns	ns	N/A	N/A	N/A	ns	ns	ns
**Rantes**	Mean	576.7	4174	1313	625.6	1911	1935	1870	2157	831.9	1189	2128	5236	3413
	Std. Deviation	172.6	3432	275.8	153.2	180	229	694.2	664.8	390.4	498.6	213.4	4556	1259
	Std. Error of Mean	86.3	1716	137.9	76.59	89.99	114.5	347.1	332.4	195.2	249.3	106.7	2278	629.5
	Compared to media control		*	ns	ns	ns	ns	ns	ns	ns	ns	ns	**	ns
	Compared to 1714.		ns	ns	ns	ns	ns	ns	N/A	N/A	N/A	ns	**	ns
**TGF-α**	Mean	2.814	8.021	1.92	0.7558	3.909	6.191	4.228	4.986	0.9797	0.9183	7.826	14.27	8.251
	Std. Deviation	4.247	1.975	0.3922	0.312	1.72	2.656	1.055	1.747	0.1346	0.6227	4.371	5.167	1.544
	Std. Error of Mean	2.123	0.9874	0.1961	0.156	0.8598	1.328	0.5277	0.8733	0.06729	0.3113	2.185	2.584	0.7718
	Compared to media control		ns	ns	ns	ns	ns	ns	ns	ns	ns	ns	****	*
	Compared to 1714.		ns	ns	ns	ns	ns	ns	N/A	N/A	N/A	ns	****	**
**TNF-β**	Mean	3.506	7.927	5.417	0.8174	4.845	8.642	5.859	5.834	1.183	1.41	7.557	11.3	7.044

		DMEM	35624100:1	35624 50:1	35624 10:1	0103 100:1	0103 50:1	0103 10:1	1714 100:1	1714 50:1	1714 10:1	AHC7 100:1	AHC7 50:1	AHC7 10:1
	Std. Deviation	4.896	1.67	1.503	0.7229	2.091	3.214	1.31	1.865	0.9218	0.8183	1.021	7.215	3.418
	Std. Error of Mean	2.448	0.8351	0.7513	0.3614	1.046	1.607	0.6551	0.9325	0.4609	0.4091	0.5103	3.608	1.709
	Compared to media control		ns	ns	ns	ns	ns	ns	ns	ns	ns	ns	**	ns
	Compared to 1714.		ns	ns	ns	ns	*	ns	N/A	N/A	N/A	ns	***	ns
**VEGF-A**	Mean	45.62	218.3	157.8	85.08	165.2	193.2	241.5	208.6	75.11	55.67	174.3	262.5	306
	Std. Deviation	28.33	69.7	24.39	31.29	32.27	33.67	62.22	49.09	52.09	49.8	19.49	128.1	146.2
	Std. Error of Mean	14.17	34.85	12.2	15.64	16.14	16.83	31.11	24.54	26.05	24.9	9.746	64.07	73.12
	Compared to media control		**	ns	ns	ns	*	**	*	ns	ns	ns	***	****
	Compared to 1714.		ns	ns	*	ns	ns	**	N/A	N/A	N/A	ns	**	****
**IL-1β**	Mean	2.295	512.4	48.41	1.982	12326	11246	1924	192.2	5.179	0.897	10497	7203	3055
	Std. Deviation	2.687	275	27.47	1.538	2610	6130	1168	86.62	3.333	0.4262	2067	2698	1322
	Std. Error of Mean	1.344	137.5	13.74	0.7691	1305	3065	584.1	43.31	1.667	0.2131	1034	1349	661.2
	Compared to media control		ns	ns	ns	****	****	ns	ns	ns	ns	****	***	ns
	Compared to 1714.		ns	ns	ns	****	****	ns	N/A	N/A	N/A	****	***	ns
**SCD40L**	Mean	92.49	193.7	169.4	91.06	143.2	164.4	218.3	194.7	119	101.3	171.7	282.6	290.9
	Std. Deviation	15.26	47.97	57.24	19.73	87.44	86.2	48.56	73.27	33.25	27.87	76.21	95.59	194.1
	Std. Error of Mean	7.629	23.98	28.62	9.864	43.72	43.1	24.28	36.64	16.63	13.93	38.11	47.8	97.03
	Compared to media control		ns	ns	ns	ns	ns	ns	ns	ns	ns	ns	*	*

		DMEM	35624100:1	35624 50:1	35624 10:1	0103 100:1	0103 50:1	0103 10:1	1714 100:1	1714 50:1	1714 10:1	AHC7 100:1	AHC7 50:1	AHC7 10:1
	Compared to 1714.		ns	ns	ns	ns	ns	ns	N/A	N/A	N/A	ns	ns	*

Supplementary Table S1 (B). MDDC cytokine secretion in response to Bifidobacteria. Statistical significance was determined using the Kruskal-Wallis test and using the ANOVA and Dunnett's multiple-comparison ****p < 0.0001, ***p < 0.001,**p < 0.01,*p < 0.05, compared to media control, or Compared to 1714.

		RPMI	35624 100:1	35624 10:1	0103 100:1	0103 10:1	1714 100:1	1714 10:1	AHC7 100:1	AHC7 10:1
**IL-10**	Mean	28.65	1999	83.17	14282	3726	571.8	57.46	20049	6188
	Std. Deviation	13.24	647.3	31.07	4724	773.8	243.8	17.92	3946	1040
	Std. Error of Mean	5.407	264.3	12.68	1929	315.9	99.53	7.315	1611	424.6
	Compared to media control		ns	ns	****	*	ns	ns	****	****
	Compared to 1714.		ns	ns	****	*	N/A	N/A	****	****
**TNF-α**	Mean	15.29	286.2	36.53	3912	1077	158.4	44.77	3702	1677
	Std. Deviation	10.15	167.4	23.86	1126	298.7	116.7	20.2	1583	797
	Std. Error of Mean	4.143	68.35	9.739	459.7	121.9	47.64	8.246	646.4	325.4
	Compared to media control		ns	ns	****	ns	ns	ns	****	**
	Compared to 1714.		ns	ns	****	ns	N/A	N/A	****	**
**EGF**	Mean	5.12	15.41	5.667	24.56	16.13	8.435	5.175	26.34	21.48
	Std. Deviation	5.928	3.968	3.68	2.025	3.895	7.096	5.229	2.913	2.702
	Std. Error of Mean	2.42	1.62	1.502	0.8266	1.59	2.897	2.135	1.189	1.103
	Compared to media control		**	ns	****	***	ns	ns	****	****
	Compared to 1714.		ns	ns	****	****	N/A	N/A	****	****
**Eotaxin**	Mean	8.76	49.75	34.8	53.28	47.36	45.2	28.03	53.48	50.62
	Std. Deviation	9.623	6.8	6.904	4.793	6.039	4.243	3.506	4.378	6.378

		RPMI	35624 100:1	35624 10:1	0103 100:1	0103 10:1	1714 100:1	1714 10:1	AHC7 100:1	AHC7 10:1
	Std. Error of Mean	3.928	2.776	2.818	1.957	2.465	1.732	1.431	1.787	2.604
	Compared to media control		****	****	****	****	****	****	****	****
	Compared to 1714.		ns	ns	ns	****	N/A	N/A	ns	****
**FGF-2**	Mean	22.61	40.78	13.76	65.15	48.45	38.87	33.81	59.12	59.04
	Std. Deviation	25.47	31.74	22.93	12.42	20.65	23.59	20.77	30.09	10.06
	Std. Error of Mean	10.4	12.96	9.363	5.069	8.432	9.631	8.481	12.29	4.108
	Compared to media control		ns	ns	*	ns	ns	ns	ns	ns
	Compared to 1714.		ns	ns	ns	ns	N/A	N/A	ns	ns
**FLT-3L**	Mean	12.44	30.35	3.053	32.53	16.77	19.63	8.726	23.31	30.2
	Std. Deviation	11.62	20.35	7.477	13.14	15.86	17.98	11.03	16.71	17.31
	Std. Error of Mean	4.743	8.309	3.053	5.363	6.476	7.339	4.501	6.823	7.069
	Compared to media control		ns	ns	ns	ns	ns	ns	ns	ns
	Compared to 1714.		ns	ns	ns	ns	N/A	N/A	ns	ns
**Fractalkine**	Mean	42.2	208	97.45	246.4	202.3	139.7	123	283.7	224.7
	Std. Deviation	38.26	52.68	54.91	38.73	41.48	59.76	64.78	40.61	47.12
	Std. Error of Mean	15.62	21.51	22.42	15.81	16.93	24.4	26.45	16.58	19.24
	Compared to media control		****	ns	****	****	**	*	****	****
	Compared to 1714.		ns	ns	**	*	N/A	N/A	****	**
**G-CSF**	Mean	34.46	197.1	49.44	5249	353.1	103.9	49.79	7782	774.4
	Std. Deviation	13.99	53.71	9.155	1120	114.6	29.63	13.41	1607	261.2
	Std. Error of Mean	5.713	21.93	3.738	457.2	46.78	12.1	5.473	656	106.6
	Compared to media control		ns	ns	****	ns	ns	ns	****	ns

		RPMI	35624 100:1	35624 10:1	0103 100:1	0103 10:1	1714 100:1	1714 10:1	AHC7 100:1	AHC7 10:1
	Compared to 1714.		ns	ns	****	ns	N/A	N/A	****	ns
**GM-CSF**	Mean	1124	737.7	923.4	1553	778	772.8	1034	1388	866.7
	Std. Deviation	655.1	499.9	575	722.9	544.7	491.6	647.6	607.2	630.8
	Std. Error of Mean	267.4	204.1	234.8	295.1	222.4	200.7	264.4	247.9	257.5
	Compared to media control		ns	ns	ns	ns	ns	ns	ns	ns
	Compared to 1714.		ns	ns	ns	ns	N/A	N/A	ns	ns
**GRO**	Mean	224.5	7878	1534	11114	7782	4837	994.1	13297	11214
	Std. Deviation	140.7	1435	663.7	1370	1643	1375	380.8	1587	1953
	Std. Error of Mean	57.45	586	271	559.4	670.8	561.2	155.4	647.9	797.4
	Compared to media control		****	ns	****	****	****	ns	****	****
	Compared to 1714.		**	ns	****	****	N/A	N/A	****	****
**IL-1α**	Mean	0.8169	4.712	1.542	31.04	6.312	2.744	3.565	14.39	7.443
	Std. Deviation	0.8548	3.409	1.276	9.755	3.816	1.227	1.802	2.805	1.856
	Std. Error of Mean	0.349	1.392	0.5208	3.983	1.558	0.501	0.7356	1.145	0.7576
	Compared to media control		ns	ns	****	ns	ns	ns	****	*
	Compared to 1714.		ns	ns	****	ns	N/A	N/A	****	ns
**IL-1RA**	Mean	37.5	152.2	58.52	428.6	219.3	106	54.04	451.3	242.8
	Std. Deviation	29.34	30.36	41.72	29.16	42.54	45.34	30.65	46.37	58.34
	Std. Error of Mean	11.98	12.39	17.03	11.9	17.37	18.51	12.51	18.93	23.82
	Compared to media control		****	ns	****	****	*	ns	****	****
	Compared to 1714.		ns	ns	****	****	N/A	N/A	****	****
**IL-2RA**	Mean	11.2	27.07	10.6	93.64	28.56	19.95	10.22	141	33.92
	Std. Deviation	7.087	7.981	6.511	42.89	7.204	16.08	7.22	75.94	8.934

		RPMI	35624 100:1	35624 10:1	0103 100:1	0103 10:1	1714 100:1	1714 10:1	AHC7 100:1	AHC7 10:1
	Std. Error of Mean	2.893	3.258	2.658	17.51	2.941	6.566	2.948	31	3.647
	Compared to media control		ns	ns	***	ns	ns	ns	****	ns
	Compared to 1714.		ns	ns	***	ns	N/A	N/A	****	ns
**IL-3**	Mean	0.3518	1.043	0.3025	2.013	0.668	0.5231	1.304	2.022	1.812
	Std. Deviation	0.5175	0.6846	0.282	0.7676	0.8263	0.7668	2.135	0.962	0.9727
	Std. Error of Mean	0.2113	0.2795	0.1151	0.3134	0.3373	0.313	0.8716	0.3927	0.3971
	Compared to media control		ns	ns	*	ns	ns	ns	*	ns
	Compared to 1714.		ns	ns	ns	ns	N/A	N/A	ns	ns
**IL-4**	Mean	1176	1028	1113	815	1007	1015	1124	806.4	940
	Std. Deviation	511.9	418	530.4	400.8	509.8	458.8	504.3	373.8	440.7
	Std. Error of Mean	209	170.6	216.6	163.6	208.1	187.3	205.9	152.6	179.9
	Compared to media control		ns	ns	ns	ns	ns	ns	ns	ns
	Compared to 1714.		ns	ns	ns	ns	N/A	N/A	ns	ns
**IL-5**	Mean	0.477	0.7671	0.2529	0.9293	0.9223	0.5009	1.222	1.21	1.194
	Std. Deviation	0.4878	0.5155	0.2331	0.7279	0.7683	0.6223	1.821	0.4652	0.9602
	Std. Error of Mean	0.1992	0.2104	0.09515	0.2972	0.3136	0.254	0.7433	0.1899	0.392
	Compared to media control		ns	ns	ns	ns	ns	ns	ns	ns
	Compared to 1714.		ns	ns	ns	ns	N/A	N/A	ns	ns
**IL-6**	Mean	984	2979	428.6	5889	5234	3203	181.2	4287	186.3
	Std. Deviation	933.8	1633	509.2	5177	2348	2059	153.6	3327	207.8
	Std. Error of Mean	381.2	666.6	207.9	2114	958.5	840.5	62.7	1358	84.83
	Compared to media control		ns	ns	**	*	ns	ns	ns	ns

		RPMI	35624 100:1	35624 10:1	0103 100:1	0103 10:1	1714 100:1	1714 10:1	AHC7 100:1	AHC7 10:1
	Compared to 1714.		ns	ns	ns	*	N/A	N/A	ns	ns
**IL-7**	Mean	22.79	81.45	47.39	96.87	81.37	67.38	39	97.11	88.03
	Std. Deviation	8.256	16.55	14.31	18.79	15.34	13.86	8.436	13.69	16.83
	Std. Error of Mean	3.37	6.755	5.841	7.671	6.264	5.659	3.444	5.588	6.87
	Compared to media control		****	*	****	****	****	ns	****	****
	Compared to 1714.		ns	ns	**	****	N/A	N/A	**	****
**IL-8**	Mean	975.1	69258	5588	78725	69194	25255	3424	70730	57970
	Std. Deviation	568	56122	2447	54922	43034	6647	770.3	29191	31926
	Std. Error of Mean	231.9	22912	998.9	22422	17569	2714	314.5	13055	14278
	Compared to media control		**	ns	**	**	ns	ns	**	*
	Compared to 1714.		ns	ns	*	**	N/A	N/A	ns	ns
**IL-9**	Mean	7.783	2.392	0.9999	11.01	6.606	2.146	1.882	9.277	6.425
	Std. Deviation	5.339	0.8731	0.7515	3.041	2.454	0.9402	1.089	1.768	1.111
	Std. Error of Mean	2.18	0.3564	0.3068	1.241	1.002	0.3838	0.4445	0.7218	0.4538
	Compared to media control		**	****	ns	ns	**	***	ns	ns
	Compared to 1714.		ns	ns	****	**	N/A	N/A	****	*
**IL-12p40**	Mean	12.6	44.74	10.14	2165	396.4	34.34	19.71	2342	1349
	Std. Deviation	8.875	38.58	11.18	2413	598.5	27.12	8.819	2455	1571
	Std. Error of Mean	3.623	15.75	4.563	985.2	244.3	11.07	3.6	1002	641.4
	Compared to media control		ns	ns	*	ns	ns	ns	*	ns
	Compared to 1714.		ns	ns	*	ns	N/A	N/A	*	ns
**IL-12p70**	Mean	1.27	5.679	2.672	47.34	36.67	6.311	2.778	56.77	132
	Std. Deviation	1.502	3.636	2.334	63.9	53.83	5.021	2.933	82.26	195.6

		RPMI	35624 100:1	35624 10:1	0103 100:1	0103 10:1	1714 100:1	1714 10:1	AHC7 100:1	AHC7 10:1
	Std. Error of Mean	0.6131	1.484	0.953	26.09	21.98	2.05	1.197	33.58	79.86
	Compared to media control		ns	ns	ns	ns	ns	ns	ns	*
	Compared to 1714.		ns	ns	ns	ns	N/A	N/A	ns	*
**IL-13**	Mean	2.973	8.703	1.885	12.18	9.889	3.673	3.455	14.26	10.92
	Std. Deviation	2.629	6.846	2.795	6.497	3.115	3.774	3.746	7.706	4.447
	Std. Error of Mean	1.073	2.795	1.141	2.652	1.272	1.541	1.529	3.146	1.816
	Compared to media control		ns	ns	*	ns	ns	ns	**	*
	Compared to 1714.		ns	ns	*	ns	N/A	N/A	**	ns
**IL-15**	Mean	2.181	5.878	2.859	11.11	9.447	1.663	5.256	10.5	7.366
	Std. Deviation	1.941	1.933	3.281	3.682	4.331	1.619	4.675	5.642	6.759
	Std. Error of Mean	0.7926	0.7892	1.34	1.503	1.768	0.6608	1.908	2.303	2.76
	Compared to media control		ns	ns	**	*	ns	ns	**	ns
	Compared to 1714.		ns	ns	**	ns	N/A	N/A	**	ns
**IL-17**	Mean	1.422	3.33	1.006	6.726	5.578	3.474	1.628	5.183	4.871
	Std. Deviation	1.047	1.045	0.9022	2.001	0.9887	1.36	1.172	0.4913	1.751
	Std. Error of Mean	0.4273	0.4266	0.3683	0.817	0.4036	0.5552	0.4785	0.2006	0.7146
	Compared to media control		ns	ns	****	****	*	ns	****	***
	Compared to 1714.		ns	ns	***	****	N/A	N/A	ns	***
**IFN-α**	Mean	9.329	47.9	14.16	65.82	55.48	36.95	15.67	67.61	63.48
	Std. Deviation	8.118	15.08	11.96	10.31	17.14	6.649	8.854	18.78	14.62
	Std. Error of Mean	3.314	6.155	4.884	4.208	6.997	2.715	3.615	7.665	5.968
	Compared to media control		****	ns	****	****	**	ns	****	****

		RPMI	35624 100:1	35624 10:1	0103 100:1	0103 10:1	1714 100:1	1714 10:1	AHC7 100:1	AHC7 10:1
	Compared to 1714.		ns	ns	**	****	N/A	N/A	**	****
**IFN-γ**	Mean	2.139	14.37	3.44	19.79	12.61	8.46	3.915	21.36	13.62
	Std. Deviation	1.478	3.89	2.335	9.138	1.826	5.288	2.789	8.491	3.821
	Std. Error of Mean	0.6032	1.588	0.9531	3.731	0.7453	2.159	1.139	3.467	1.56
	Compared to media control		***	ns	****	**	ns	ns	****	**
	Compared to 1714.		ns	ns	**	*	N/A	N/A	***	*
**IP-10**	Mean	9.418	137.9	51.52	20	50.68	106.8	32.95	16.15	35.12
	Std. Deviation	9.326	152.3	44.49	14.58	42.78	149.9	32.95	10.11	25.52
	Std. Error of Mean	3.807	62.18	18.16	5.953	17.46	61.18	13.45	4.129	10.42
	Compared to media control		*	ns	ns	ns	ns	ns	ns	ns
	Compared to 1714.		ns	ns	ns	ns	N/A	N/A	ns	ns
**MCP-1**	Mean	162.2	11121	560.7	6363	6549	3824	521.4	7971	16676
	Std. Deviation	166.6	5589	804.8	5726	5493	5967	797.1	6869	4421
	Std. Error of Mean	68.02	2282	328.6	2338	2242	2436	325.4	2804	1805
	Compared to media control		**	ns	ns	ns	ns	ns	*	****
	Compared to 1714.		ns	ns	ns	ns	N/A	N/A	ns	****
**MCP-3**	Mean	3.487	13.71	7.438	11.56	14.98	4.403	6.692	15.57	37.58
	Std. Deviation	8.542	22.4	11.56	18.17	16.51	10.78	10.49	24.5	58.23
	Std. Error of Mean	3.487	9.144	4.718	7.418	6.741	4.403	4.285	10	23.77
	Compared to media control		ns	ns	ns	ns	ns	ns	ns	ns
	Compared to 1714.		ns	ns	ns	ns	N/A	N/A	ns	ns
**MDC**	Mean	936.8	3135	1352	13136	3632	2316	1481	10035	4412
	Std. Deviation	1199	4949	2074	13514	5095	3808	2466	14119	6643

		RPMI	35624 100:1	35624 10:1	0103 100:1	0103 10:1	1714 100:1	1714 10:1	AHC7 100:1	AHC7 10:1
	Std. Error of Mean	489.3	2021	846.5	5517	2080	1555	1007	5764	2712
	Compared to media control		ns	ns	*	ns	ns	ns	ns	ns
	Compared to 1714.		ns	ns	ns	ns	N/A	N/A	ns	ns
**MIP-1α**	Mean	10.24	1465	27.34	12249	3830	280.7	30.24	11104	6998
	Std. Deviation	7.15	1174	16.07	5305	2465	216	22.52	2224	3451
	Std. Error of Mean	2.919	479.2	6.559	2166	1006	88.18	9.194	908	1409
	Compared to media control		ns	ns	****	ns	ns	ns	****	****
	Compared to 1714.		ns	ns	****	ns	N/A	N/A	****	****
**MIP-1β**	Mean	63.14	2525	185.5	13790	4052	920.4	173.9	20607	7652
	Std. Deviation	32.84	915.7	82.02	6848	1505	469.5	90.89	13677	3150
	Std. Error of Mean	13.41	373.8	33.48	2796	614.6	191.7	37.11	5584	1286
	Compared to media control		ns	ns	***	ns	ns	ns	****	ns
	Compared to 1714.		ns	ns	***	ns	N/A	N/A	****	ns
**PDGF-AA**	Mean	4.886	9.533	5.364	27.4	11.61	7.818	6.642	26.78	16.79
	Std. Deviation	3.755	5.55	4.123	9.162	5.295	6.001	5.797	6.786	7.554
	Std. Error of Mean	1.533	2.266	1.683	3.741	2.162	2.45	2.367	2.77	3.084
	Compared to media control		ns	ns	****	ns	ns	ns	****	*
	Compared to 1714.		ns	ns	****	ns	N/A	N/A	****	*
**PDGF-BB**	Mean	1.411	17.17	10.07	16.54	22.14	2.515	8.579	14.66	8.867
	Std. Deviation	3.456	22.46	24.67	24.61	35.2	5.811	21.02	19.75	21.37
	Std. Error of Mean	1.411	9.168	10.07	10.05	14.37	2.372	8.579	8.065	8.722
	Compared to media control		ns	ns	ns	ns	ns	ns	ns	ns

		RPMI	35624 100:1	35624 10:1	0103 100:1	0103 10:1	1714 100:1	1714 10:1	AHC7 100:1	AHC7 10:1
	Compared to 1714.		ns	ns	ns	ns	N/A	N/A	ns	ns
**Rantes**	Mean	3.873	76.27	7.151	621.7	170.4	27.26	7.801	595.4	237.2
	Std. Deviation	3.289	47.71	5.849	216.6	72	21.83	5.595	272.8	119.5
	Std. Error of Mean	1.343	19.48	2.388	88.45	29.4	8.914	2.284	111.4	48.78
	Compared to media control		ns	ns	****	ns	ns	ns	****	*
	Compared to 1714.		ns	ns	****	ns	N/A	N/A	****	*
**TGF-α**	Mean	7.868	49.97	15.78	140.5	55.26	32.59	14.68	162	75.18
	Std. Deviation	2.392	20.25	6.749	48.06	17.86	15.19	7.443	63.31	28.18
	Std. Error of Mean	0.9767	8.265	2.755	19.62	7.293	6.2	3.039	25.85	11.5
	Compared to media control		ns	ns	****	ns	ns	ns	****	**
	Compared to 1714.		ns	ns	****	ns	N/A	N/A	****	**
**TNF-β**	Mean	1.595	4.29	0.7111	7.135	9.213	3.242	0.4756	8.865	6.826
	Std. Deviation	2.471	2.588	1.036	1.435	3.239	4.17	0.8869	2.038	2.993
	Std. Error of Mean	1.009	1.057	0.4229	0.5858	1.322	1.702	0.3621	0.8321	1.222
	Compared to media control		ns	ns	**	****	ns	ns	****	**
	Compared to 1714.		ns	ns	ns	****	N/A	N/A	**	***
**VEGF-A**	Mean	25.63	106	30.04	143	94.48	55.77	46.35	219.4	127.6
	Std. Deviation	29.87	76.75	32.62	31.05	51.6	56.67	30.08	55.98	39.26
	Std. Error of Mean	12.2	31.33	13.32	12.68	21.07	23.14	12.28	22.85	16.03
	Compared to media control		*	ns	***	ns	ns	ns	****	**
	Compared to 1714.		ns	ns	*	ns	N/A	N/A	****	*
**IL-1β**	Mean	2.039	11.68	2.821	83.05	21.9	7.018	2.709	46.79	23.01
	Std. Deviation	1.663	3.646	1.953	37.96	8.834	6.524	2.313	19.76	11.22

		RPMI	35624 100:1	35624 10:1	0103 100:1	0103 10:1	1714 100:1	1714 10:1	AHC7 100:1	AHC7 10:1
	Std. Error of Mean	0.6787	1.489	0.7973	15.5	3.606	2.664	0.9441	8.065	4.581
	Compared to media control		ns	ns	****	ns	ns	ns	****	ns
	Compared to 1714.		ns	ns	****	ns	N/A	N/A	***	ns
**SCD40L**	Mean	17.74	33.2	18.09	39.94	32.88	42.95	16.64	44.04	32.24
	Std. Deviation	15.46	12.39	12.5	9.639	22.33	23.5	13.28	13.14	25.95
	Std. Error of Mean	6.312	5.059	5.102	3.935	9.117	9.594	5.422	5.364	10.59
	Compared to media control		ns	ns	ns	ns	ns	ns	ns	ns
	Compared to 1714.		ns	ns	ns	ns	N/A	N/A	ns	ns

Supplementary Table S1 (C). MDDC cell surface expression of costimulatory and inhibitory molecules following exposure to four bifidobacterial strains. Statistical significance was determined using the Kruskal-Wallis test and using the ANOVA and Dunnett's multiple-comparison ****p < 0.0001, ***p < 0.001, **p < 0.01, *p < 0.05, compared to media control or Compared to 1714.

		RPMI	35624 100:1	35624 10:1	0103 100:1	0103 10:1	1714 100:1	1714 10:1	AHC7 100:1	AHC7 10:1
**CD86**	Mean	8.183	14.7	6.3	62.25	26.33	9.35	6.117	47.47	27.4
	Std. Deviation	3.366	11.96	4.559	18.28	20.03	7.163	4.502	25.44	24.4
	Std. Error of Mean	1.374	4.883	1.861	7.462	8.179	2.924	1.838	10.39	9.962
	Compared to media control		ns	ns	****	ns	ns	ns	***	ns
	Compared to 1714.		ns	ns	****	ns	N/A	N/A	***	ns
**CD80**	Mean	3.133	15.38	4.983	33.17	15.83	9.033	3.433	27.88	24.38
	Std. Deviation	1.74	7.68	2.805	16.24	9.706	4.423	2.317	15.76	14.12

		RPMI	35624 100:1	35624 10:1	0103 100:1	0103 10:1	1714 100:1	1714 10:1	AHC7 100:1	AHC7 10:1
	Std. Error of Mean	0.7102	3.136	1.145	6.631	3.962	1.805	0.9461	6.432	5.765
	Compared to media control		ns	ns	****	ns	ns	ns	***	**
	Compared to 1714.		ns	ns	***	ns	N/A	N/A	*	**
**HLA-DR**	Mean	19.42	21.1	21.37	24.62	23	20.33	19.58	23.48	23.75
	Std. Deviation	5.006	5.217	5.756	5.529	7.317	3.966	4.16	5.694	5.895
	Std. Error of Mean	2.044	2.13	2.35	2.257	2.987	1.619	1.698	2.325	2.406
	Compared to media control		ns	ns	ns	ns	ns	ns	ns	ns
	Compared to 1714.		ns	ns	ns	ns	N/A	N/A	ns	ns
**DCIR**	Mean	43.25	47.07	42.47	8.45	45.12	49.75	43.32	26.07	30.62
	Std. Deviation	19.04	23.32	19.75	10.02	25.03	23.75	19.71	22.02	20.7
	Std. Error of Mean	7.774	9.519	8.062	4.092	10.22	9.697	8.046	8.988	8.451
	Compared to media control		ns	ns	*	ns	ns	ns	ns	ns
	Compared to 1714.		ns	ns	**	ns	N/A	N/A	ns	ns
**IL-T3**	Mean	6.525	6.65	8.767	0.4833	9.067	9.45	9.283	1.05	5.433
	Std. Deviation	5.984	4.452	6.458	0.5947	10.04	5.933	7.562	1.555	5.487
	Std. Error of Mean	2.443	1.817	2.636	0.2428	4.098	2.422	3.087	0.635	2.24
	Compared to media control		ns	ns	ns	ns	ns	ns	ns	ns
	Compared to 1714.		ns	ns	ns	ns	N/A	N/A	ns	ns

		RPMI	35624 100:1	35624 10:1	0103 100:1	0103 10:1	1714 100:1	1714 10:1	AHC7 100:1	AHC7 10:1
**PD-L1**	Mean	6.433	12.42	9.083	24.83	17.12	12.48	8.817	25.68	17.05
	Std. Deviation	6.076	6.786	5.278	13.99	13.67	6.502	4.515	11.42	11.31
	Std. Error of Mean	2.48	2.77	2.155	5.712	5.582	2.655	1.843	4.661	4.617
	Compared to media control		ns	ns	*	ns	ns	ns	**	ns
	Compared to 1714.		ns	ns	ns	ns	N/A	N/A	ns	ns
**PD-L2**	Mean	28.53	32.15	30.5	77.83	51.27	30.22	31.67	73.47	54.33
	Std. Deviation	11.6	8.049	10.9	15.96	15.55	8.087	11.71	17.33	18.59
	Std. Error of Mean	4.736	3.286	4.448	6.516	6.347	3.302	4.779	7.074	7.587
	Compared to media control		ns	ns	****	*	ns	ns	****	*
	Compared to 1714.		ns	ns	****	ns	N/A	N/A	****	*
