# Supplementary material for: Complementary immunoregulatory effects of Bifidobacterium longum 1714TM associated exopolysaccharide and tryptophan metabolism
Source: Curr Res Microb Sci. 2025 Sep 28;9:100481. doi: 10.1016/j.crmicr.2025.100481 (PMC12546897; doi:10.1016/j.crmicr.2025.100481)
Supplement: Supplementary file 7 [file mmc7.pdf]

Supplementary Table S2. Components of *B. longum* 1714 genome corresponding to color codes in the EPS cluster.

Locus tag (1714)	Function	No. of amino acids	No. of transmembrane helices (TMHMM)	Motifs & Domains (EMBL)
0371	priming glycosyl transferase CpsD	531	5	Bacterial sugar transferase
0372	conserved hypothetical protein	644	1	
0373	tyrosine kinase	501	2	• Chain length determinant protein<br>• CobQ/CobB/MinD/ParA nucleotide binding domain
0374	hypothetical glycosyl transferase, group 1family protein	384	0	• Domain of unknown function (DUF1972)<br>• Glycosyl transferases group 1
0375	putative glycosyltransferase protein	447	0	Glycosyl transferases group 1
0376	UDP-glucuronate 5'-epimerase	354	0	NAD dependent epimerase/dehydratase family
0377	UDP-glucose 6-dehydrogenase	416	0	• UDP-glucose/GDP-mannose dehydrogenase family, NAD binding domain<br>• UDP-glucose/GDP-mannose dehydrogenase family, central domain<br>• UDP-glucose/GDP-mannose dehydrogenase family, UDP binding domain
0378	hypothetical glycosyl transferase, group 1 family protein	354	0	Glycosyl transferases group 1

0379	NAD dependent epimerase/dehydratase	276	0	NAD dependent epimerase/dehydratase family
0380	acetyltransferase	194	0	Bacterial transferase hexapeptide (six repeats)
0381	putative glycosyltransferase	96	0	
0382	Possible polymerase involved in polysaccharide biosynthesis	447	11	O-Antigen ligase
0383	Conserved hypothetical protein Eps9K	310	0	
0384	Flippase protein involved in polysaccharide biosynthesis	481	14	Polysaccharide biosynthesis protein
0384A	Acetyltransferase	126	0	Bacterial transferase hexapeptide (six repeats)
0386	NAD-dependent epimerase/dehydratase familyprotein, putative	383	0	NAD dependent epimerase/dehydratase family
0392	dTDP-glucose 4,6-dehydratase	340	0	NAD dependent epimerase/dehydratase family
0393	bifunctional dTDP-4-dehydrorhamnose 3,5-epimerase/dTDP-4-dehydrorhamnose reductase	362	0	• dTDP-4-dehydrorhamnose 3,5-epimerase<br>• RmlD substrate binding domain
0394	glucose-1-phosphate thymidylyltransferase	299	0	Nucleotidyl transferase
0399	hypothetical phosphotyrosine protein phosphatase	186	0	Low molecular weight phosphotyrosine protein phosphatase
