# Supplementary material for: Complementary immunoregulatory effects of Bifidobacterium longum 1714TM associated exopolysaccharide and tryptophan metabolism
Source: Curr Res Microb Sci. 2025 Sep 28;9:100481. doi: 10.1016/j.crmicr.2025.100481 (PMC12546897; doi:10.1016/j.crmicr.2025.100481)
Supplement: Supplementary file 8 [file mmc8.pdf]

Supplementary Table S3 (A). Gut Brain modules and function identified in *B. longum* 1714.

System	Function	Pathway	Predicted EC
**Isovaleric acid synthesis II (KADC pathway)**	SCFA	Leucine-derived	1.2.1.3, 2.6.1.42
**Tryptophan synthesis**	Amino acid	Tryptophan-derived	2.4.2.18,4.1.1.48,4.2.3.4,2.7.1.71,2.5.1.19,5.3.1.24,1.1.1.25,4.1.2.8,4.2.3.5,4.1.3.27,4.2.1.10,2.5.1.54
**Glutamate synthesis I/II**	Inhibitory neurotransmitter	Glutamate-derived	1.4.1.3, 1.4.1.4, 1.4.7.1, 3.5.1.2, 1.4.1.13,
**ClpB (ATP-dependent chaperone protein)**	Chaperone	ClpB	3.4.21.92
**Quinolinic acid degradation**	Excitotoxic, neuroinflammatory	Tryptophan	2.4.2.19, 2.7.7.18,6.3.5.1
**S-Adenosylmethionine (SAM) synthesis**	Methyltransferase, anti-inflammatory	Methionine-derived	2.5.1.6
**Acetate synthesis I**	SCFA	Acetate	2.7.2.1, 2.7.2.15, 6.2.1.13, 2.3.1.54, 2.3.1.8
**Tryptophan degradation**	Amino acid	Tryptophan-derived	3.5.99.10,4.1.99.1, 1.1.1.110,2.6.1.27, 2.6.1.28,1.1.1.190,4.1.1.74

Supplementary Table S3 (B). BLAST results from comparison of NCC2705 tryptophan biosynthesis cluster to *B. longum* 1714.

Step	Gene ID in NCC2705	Description	1714 gene locus tag ID	% identity	Predicted EC
trpE	BL_RS01930	anthranilate synthase subunit TrpE	27855_Genome14_00178	100	4.1.3.27
trpD_1	BL_RS06715	glutamine amidotransferase of anthranilate synthase	27855_Genome14_01504	100	4.1.3.27
trpD_2	BL_RS01380	anthranilate phosphoribosyltransferase	27855_Genome14_00124	97.7	2.4.2.18
PRAI	BL_RS00735	phosphoribosylanthranilate isomerase	27855_Genome14_00009	100	5.3.1.16
IGPS	BL_RS02310	indole-3-glycerol phosphate synthase	27855_Genome14_00313	100	4.2.1.20
trpA	BL_RS02305	indoleglycerol phosphate aldolase	27855_Genome14_00312	100	4.2.1.20
trpB	BL_RS02310	tryptophan synthase	27855_Genome14_00313	100	4.2.1.20
