# Supplementary material for: Complementary immunoregulatory effects of Bifidobacterium longum 1714TM associated exopolysaccharide and tryptophan metabolism
Source: Curr Res Microb Sci. 2025 Sep 28;9:100481. doi: 10.1016/j.crmicr.2025.100481 (PMC12546897; doi:10.1016/j.crmicr.2025.100481)
Supplement: Supplementary file 9 [file mmc9.pdf]

Supplementary Table S4. Plasma metabolites. Statistical significance for the metabolite was determined using the ANOVA and Dunnett's multiple comparison *p < 0.05, vs. Week 0.

		***B. longum* 1714**			**Placebo**		
		**Week 0**	**Week 4**	**Week 8**	**Week 0**	**Week 4**	**Week 8**
**Tryptophan mM**	Mean	**8.359**	8.635	8.819	**9.393**	9.028	9.519
	Std. Deviation	1.563	1.841	1.349	1.918	1.696	2.183
	Std. Error of Mean	0.3127	0.3682	0.2699	0.3391	0.2999	0.3860
			ns	*		ns	ns
**Serotonin mM**	Mean	**3.201**	2.841	3.118	**1.363**	1.336	1.300
	Std. Deviation	10.71	9.906	9.323	3.569	5.274	4.509
	Std. Error of Mean	2.142	1.981	1.865	0.6212	0.9180	0.7849
			ns	ns		ns	ns
**Kynurenine mM**	Mean	**0.1496**	0.1456	0.1556	**0.1703**	0.1639	0.1664
	Std. Deviation	0.03846	0.03056	0.03686	0.04792	0.04190	0.04197
	Std. Error of Mean	0.007692	0.006112	0.007373	0.008343	0.007294	0.007306
			ns	ns		ns	ns
**Kynurenic acid mM**	Mean	**0.005073**	0.005366	0.006360	**0.006333**	0.005595	0.006182
	Std. Deviation	0.002306	0.002939	0.003936	0.003391	0.002264	0.003358
	Std. Error of Mean	0.0003602	0.0004590	0.0007871	0.0005232	0.0003493	0.0005846
			ns	*		ns	ns
**Quinolinic acid (Area)**	Mean	**97920**	103058	110337	**115329**	105579	97961
	Std. Deviation	26466	29791	28963	37897	23952	23013
	Std. Error of Mean	5293	5958	5793	6597	4170	4006
			ns	ns		ns	0.0633

		B. longum 1714			Placebo		
		Week 0	Week 4	Week 8	Week 0	Week 4	Week 8
**DL-Indole-3-lactic acid (Area)**	Mean	**2869541**	3167015	2812281	**3274410**	3468590	3274883
	Std. Deviation	754364	1284677	906996	1333170	1295874	1080308
	Std. Error of Mean	150873	256935	181399	232075	225583	188057
			ns	ns		ns	ns
**Indole-3-propionic acid (Area)**	Mean	**749020**	860156	637480	**714579**	751843	806733
	Std. Deviation	573960	1034997	382117	344320	372299	429420
	Std. Error of Mean	114792	206999	76423	59938	64809	74753
			ns	ns		ns	ns
**Indole-3-acetic acid (Area)**	Mean	**21850878**	21936003	18815950	**18110492**	17346802	18510266
	Std. Deviation	12878071	11116991	8432679	5052368	7033844	8050086
	Std. Error of Mean	2575614	2223398	1686536	879504	1224435	1401340
			ns	ns		ns	ns
**n-acetyl-5-hydroxytryptamine (Area)**	Mean	**2212426**	2076604	2163478	**2611315**	2520960	2338321
	Std. Deviation	1355220	1283963	1315371	1465645	1373546	1478244
	Std. Error of Mean	271044	256793	263074	255136	239104	257329
			ns	ns		ns	ns
**Indole (Area)**	Mean	**836336**	907264	875274	**878107**	897803	860350
	Std. Deviation	239314	233192	298854	293878	297470	273911
	Std. Error of Mean	47863	46638	59771	51158	51783	47682
			ns	ns		ns	ns
**5-Methoxytryptophan (Area)**	Mean	**1672759**	1768907	1815775	**1738729**	1769773	1738482
	Std. Deviation	291889	402227	425719	440926	385820	382615
	Std. Error of Mean	58378	80445	85144	75618	66168	65618

		B. longum 1714			Placebo		
		**Week 0**	**Week 4**	**Week 8**	**Week 0**	**Week 4**	**Week 8**
			ns	ns		ns	ns
**5-methyl-tryptophan (Area)**	Mean	**310518**	323031	241247	**260801**	285343	261357
	Std. Deviation	220627	296616	107530	122099	165204	103619
	Std. Error of Mean	44125	59323	21506	21255	28758	18038
			ns	ns		ns	ns
**4-Indolecarbaldehyde (Area)**	Mean	**5573215**	5941584	5719020	**5646951**	5786537	6157593
	Std. Deviation	1733042	1747728	1189546	1265233	1375899	2136327
	Std. Error of Mean	346608	349546	237909	220249	239513	371887
			ns	ns		ns	ns
